# Lifecycle evaluation of medical devices: supporting or jeopardizing patient outcomes? A comparative analysis of evaluation models

**DOI:** 10.1017/S026646232300274X

**Published:** 2024-01-05

**Authors:** Kathleen R. Harkin, Jan Sorensen, Steve Thomas

**Affiliations:** 1 Centre for Health Policy and Management, Trinity College Dublin (TCD), Dublin, Ireland; 2 RCSI Healthcare Outcomes Research Centre, School of Population Health, Royal College of Surgeons in Ireland (RCSI), Dublin, Ireland

**Keywords:** medical devices, conceptual models, evaluation, lifecycle, review, qualitative synthesis, evidence

## Abstract

**Objectives:**

Lack of evidence regarding safety and effectiveness at market entry is driving the need to consider adopting a lifecycle approach to evaluating medical devices, but it is unclear what lifecycle evaluation means. This research sought to explore the tacit meanings of “lifecycle” and “lifecycle evaluation” as embodied within evaluation models/frameworks used for medical devices.

**Methods:**

Drawing on qualitative evidence synthesis methods and using an inductive approach, novel methods were developed to identify, appraise, analyze, and synthesize lifecycle evaluation models used for medical devices. Data was extracted (including purpose; audience; characterization; outputs; timing; and type of model) from key texts for coding, categorization, and comparison, exploring embodied meaning across four broad perspectives.

**Results:**

Fifty-two models were included in the synthesis. They demonstrated significant heterogeneity of meaning, form, scope, timing, and purpose. The “lifecycle” may represent a single stage, a series of stages, a cycle of innovation, or a system. “Lifecycle evaluation” focuses on the overarching goal of the stakeholder group, and may use a single or repeated evaluation to inform decision-making regarding the adoption of health technologies (Healthcare), resource allocation (Policymaking), investment in new product development or marketing (Trade and Industry), or market regulation (Regulation). The adoption of a lifecycle approach by regulators has resulted in the deferral of evidence generation to the post-market phase.

**Conclusions:**

Using a “lifecycle evaluation” approach to inform reimbursement decision-making must not be allowed to further jeopardize evidence generation and patient safety by accepting inadequate evidence of safety and effectiveness for reimbursement decisions.

## Introduction

Patients and healthcare payers value medical devices that are safe and effective. However, there is very little direct clinical evidence regarding the safety and efficacy of most medical devices when they first enter the market or even at the point of reimbursement (1–7). This has prompted many stakeholders to consider applying a lifecycle approach to evaluation with the aim of improving the evidence base over time.

However, the “medical device lifecycle” is a poorly defined concept and there is no consensus regarding what it encompasses nor regarding what is meant by lifecycle evaluation (8–11). Instead, their meaning is assumed, yet implicitly embodied within the evaluation frameworks being used.

Without clear definitions, different people assume different meanings and use these concepts for different purposes. Indeed, lifecycle evaluation may mean generating better evidence over time, but equally, it may mean accepting limited or poor-quality evidence early in the lifecycle based on the premise that more evidence will become available later (12). Unfortunately, such differences may impact patient safety if they are not made explicit. For example, assuming that a device has been proven to be safe and effective before being marketed means patient outcomes may not be analyzed to the extent that they would be if it were known that this is frequently untrue, thereby, delaying the detection of harmful devices (5;13–15). Therefore, it is necessary to understand what different people mean and understand by these concepts and how they use them.

## Objectives

### Aim

To explore the different meanings of “medical device lifecycle” and “lifecycle evaluation” as embodied within the evaluation models used by different actors involved with medical devices, and the potential impact that differences may have on patient safety.

## Methods

The unit of analysis for this literature review is the model rather than the articles. Similar to Michie, Stralen, and West, we use the term “model” to mean ““a hypothetical description of a complex entity or process.”” (16), which in this study denotes any approach, framework, model or theory utilized to evaluate medical devices across their lifespan (Supplementary Material 1). For the sake of brevity, throughout this article the terms “model” or “conceptual model” will be used to encompass the entire range of theoretical or conceptual approaches, models, and frameworks.

The included models were drawn from diverse disciplines and, consequently, demonstrated extensive heterogeneity, requiring novel methods for analysis and synthesis. These are briefly described here using the ENTREQ guidelines (17), with more detail provided where indicated in the Supplementary Materials.

### Synthesis Methodology

Using an inductive qualitative approach, models were synthesized using analytic techniques drawn from grounded theory, qualitative content analysis, thematic analysis, and meta-ethnography (18–24).

### Approach to Searching

Due to the unexpectedly large number of lifecycle evaluation approaches found in the literature, and drawing on methods described previously, a pragmatic approach to searching was adopted (25–27). Indeed, complementary and snowball techniques have previously been found to be more effective and efficient at identifying relevant material when the topic is complex and multidisciplinary (26;27). Seminal texts and models were identified through scoping searches conducted in biomedical and multidisciplinary bibliographic databases, the Internet, and specific Web sites using keywords and phrases covering the concepts “medical device,” “lifecycle,” and “model.” Snowball searching (i.e., searching references, references of references, and citations) of seminal texts was employed to identify additional models. *Electronic Search strategy*: (Supplementary Material 2).

### Data Sources

Systematic scoping searches: PubMed, EMBASE, and the Cochrane Library. Pragmatic searches: the internet, Google Scholar, and JSTOR.

### Inclusion Criteria

Any lifecycle model that may be applied to a medical device (however, that may be conceptualized, e.g., as a product, industry, innovation, therapy, technology, intervention, software or hardware, or as a specific type of device) (Supplementary Material 1) covering several aspects or stages of a device’s lifetime was eligible for inclusion (Supplementary Material 3). Models devised solely for evaluating medicines were excluded, because their lifecycle stages differ considerably from medical devices, posing different problems and being subject to different requirements.

### Study Screening Methods

Articles were systematically screened in phases against the inclusion and exclusion criteria (Supplementary Material 4). Initial screening was conducted concurrently with searching. Potentially relevant texts appearing to take a lifecycle approach were identified and saved in a document archive.

### Model Selection

Documents in the archive were systematically screened against the inclusion criteria for data extraction into an Excel database, where they were then screened against a selection algorithm to select one representative text per model, with the remainder serving as reference materials. Searching, screening, selection, and data extraction iterated with data analysis. Selection ceased when data saturation had been reached (i.e., no significantly different models or new themes were emerging). Potentially eligible models identified subsequently were saved for triangulation (Supplementary Materials 3 and 4).

### Model and Study Characteristics

The characteristics of the included models and their key reference texts are summarized in Table 2 (Supplementary Materials 8 and 9).

### Appraisal Rationale

Quality appraisal (QA), though its use in qualitative research is contested (28), serves to deepen researchers’ understanding of the data being examined, making the QA process valuable for increasing insight (29). *Appraisal items*: No QA criteria have been universally accepted for appraising conceptual models. We adopted those used by D’Amour et al. (30), assessing whether a model was underpinned by theory, empirical data, and/or an explicit literature review strategy, assigning a score of one for each criterion met. *Appraisal process*: Models were appraised rather than articles, therefore, we drew on additional texts for the appraisal process since criteria may be met in different sources. *Appraisal results* are summarized in Table 2 (Supplementary Material 7). No model was excluded based on its score.

### Data Extraction

Extracted data included study characteristics of reference texts; the model’s purpose, perspective, audience, characterization, scope, stages covered, factors included, focus of interest, representation (e.g., diagrams, graphs, mathematical equations, or textual descriptions), level of application, timepoints for analysis, process definition; and the quality criteria. Data were extracted primarily from the reference texts, but supplemented from others. *Software*: Word and Excel 2016 for Mac (Version 16.70).

### Number of Reviewers

This study was conducted as part of a PhD, therefore, one reviewer (KH) conducted all aspects of searching, screening, selection, data extraction, quality appraisal, analysis, and synthesis, with supervision, oversight, discussion, reflection, and review by two supervisors (ST and JS).

### Coding

The unit of analysis was the model, consequently, coding focused on the data extracted from the documents. Each model was categorized into one of four broad perspectives (Supplementary Material 5). As analysis progressed extracted data was coded into increasingly broad and more abstract categories, enabling comparison within and across categories. Summaries of each model were prepared, and visual tools created (Analysis cards) to enable a visual comparison of the models.

### Derivation of Themes

Using the constant comparative method, together with reflective writing, which surfaced higher-order patterns in the data, themes relevant to patient safety were derived. *Quotations* are used to support the findings.

### Positionality and Reflexivity

The researchers come from a medical, Health Technology Assessment (HTA), and/or policy background, mostly, from a positivist/postpositivist paradigm, but take a pragmatic worldview. We are interested in patient outcomes and healthcare efficiency. Consequently, personal professional perspectives and pre-conceptions were bracketed during the interpretive process and an inductive approach was taken to ground the analysis in the data (31, p. 27, 193–195).

### Trustworthiness

To ensure researcher consistency, a system of double-entry, comparison, and validation of analytic data was developed. The document archive and Excel database provide a research audit trail, with Supplementary Materials ensuring transparency.

### Synthesis Output

Several syntheses were created, including a comparative analysis of the models, the development of a model typology, and a thematic analysis. This article reports on the comparative analysis.

### Reporting

The findings are presented in three sections, with a brief overview first, followed by a tabular and narrative description of the meanings attributed to “medical device lifecycle” and “lifecycle evaluation.” The discussion explores implications, whilst the conclusion sets out the key issues and potential solutions.

## Findings

Fifty-two models were included in the synthesis, with 51 key texts, drawn from multiple different perspectives, and organized into four broad categories for comparison. Nine models were derived from Healthcare (HC), 16 from Policymaking (Policy), eight from Regulation (Reg), and 19 from Trade and Industry (T&I). Table 1 lists the models according to their perspectives, providing abbreviated names and the reference numbers for key texts, with their references provided in Supplementary Material 6.

**Table 1.** List of models included in the synthesis

Model name	*Abbreviated name*, *Author(s) of Reference text, Publication Year* (Supplemental Reference-number)^Symbol for Broad Perspective assigned to^
New Product Development	***Baldock-NPD,** Baldock, 1960* (SR-1)**™**
Life of an innovation	***DOI,** Rogers, 1962* (SR-2)^**Π**^
Bass Model	***Bass,** Bass, 1969* (SR-3)**™**
Product Life Cycle	***PLC,** Levitt, 1965* (SR-4)**™**
7 stages in the career of a medical innovation	***7Sm-IC,** McKinlay, 1981* (SR-5)^**Π**^
Life history and routinization of an innovation	***IRP,** Yin, 1981* (SR-6)^**Π**^
New Product Process	***BAH-NPD,** Booz, Allen & Hamilton, 1982* (SR-7)**™**
Business Lifecycle (High-technology ventures)	***BLC,** Galbraith, 1982* (SR-8)**™**
Industry Life Cycle	***ILC,** Gort & Klepper, 1982* (SR-9)**™**
New Product Development Process	***CK-NPD,** Cooper & Kleinschmidt, 1986* (SR-10)**™**
A diffusion theory model of adoption and substitution for successive generations of high-technology products	***Norton-Bass,** Norton & Bass, 1987* (SR-11)**™**
Stage-gate system (for new product development)	***SG-CK-NPD,** Cooper, 1990* (SR-12)**™**
Technology Adoption Life Cycle (High-Tech Marketing Model)	***TALC,** Moore, 2001* (SR-13)**™**
Generalized Bass Model	***G-Bass-M,** Bass, Krishnan, & Jain, 1994* (SR-14)**™**
Technology Readiness Levels	***TRL,** Mankins, 1995* (SR-15)^**Π**^
Medical Device Design and Development process	***MDDP,** FDA, 1997* (SR-16)^**®**^
VA Technology Transfer Process	***VA-NPD,** Sheredos & Cupo, 1997* (SR-17)^**Ψ**^
A framework of iterative economic evaluation	***4S-IEE,** Sculpher, Buxton, & Drummond, 1997* (SR-18)^**Π**^
RE-AIM Framework	***RE-AIM,** Glasgow, Vogt, & Boles, 1999* (SR-19)^**Π**^
Health Care Technology Life Cycle	***HCTLC,** Cheng, 2003* (SR-20)^**®**^
Major phases in the life span of a medical device	***MDLS,** Cheng, 2003* (SR-20)^**®**^
A systems-based user-centered approach to healthcare design	***SUHCD,** Clarkson et al., 2004* (SR-21)^**Ψ**^
Conceptual Model for Considering the Determinants of Diffusion, Dissemination, and Implementation of Innovations in Health Service Delivery and Organizations	***DDDII,** Greenhalgh et al., 2004* (SR-22)^**Π**^
Total Product Lifecycle (TPLC)	***TPLC,** Feigal Jr. for the Institute of Medicine, 2010* (SR-23)^**®**^
Technology adoption lifecycle Conceptual attractor & H Framework	***TALC-CAHF,** Meade & Rabelo, 2004* (SR-24)**™**
Equipment Life Cycle	***ELC,** Worm (THET), 2015* (SR-25)^**Ψ**^
Technology risk and readiness assessment framework	***IRM-TRL,** Mankins, 2009* (SR-26)^**Π**^
IDEAL Framework	***IDEAL,** McCulloch et al., 2009* (SR-27)^**Ψ**^
Industrial Emergence Framework	***IEF,** Phaal et al., 2009* (SR-28)**™**
Medical device design and development stage-gate process	***SG-MDDP,** Pietzsch et al., 2009* (SR-29)**™**
The Innovation Life Cycle	***IC+,** Croslin, 2010* (SR-30)**™**
Lifecycle of Technology	***TLC,** Mytton et al., 2010* (SR-31)^**Π**^
EAES guideline on methodology of innovation management in endoscopic surgery	***EIM-2DA,** Neugebauer & Becker et al., 2010* (SR-32)^**Ψ**^
New Product Development Framework	***Bhuiyan-NPD,** Bhuiyan, 2011* (SR-33)**™**
Conceptual Framework of the University Spin-off Venturing Process	***USVP,** Rasmussen, 2011* (SR-34)**™**
Medical device life-cycle	***MDLC,** Velazquez-Berumen, 2011* (SR-35)**^**Π**^**
Health Product Vigilance Framework	***HCanada-MDRegLC,** Health Canada, 2013* (SR-36)^**®**^
The Innovation Cycle (CIRAS)	***IC,** CIRAS, 2013* (SR-37)**™**
The Innovation Cycle (WW)	***WW-IC,** Wright & Weinstein, 2013* (SR-38)^**Ψ**^
The integration of risk management process with the lifecycle of MD software	***IRM-SaMDDP,** Pecoraro & Luzi, 2017* (SR-39)**™**
Treatment “lifecycle” framework	***RxLCF,*** *Provoost et al., 2014* (SR-40)^**Ψ**^
Product Innovation Life Cycle	***PILC,** Baeyens, 2016* (SR-41)^**Π**^
IDEAL-D Framework	***IDEAL-D,*** *Pennell et al., 2016* (SR-42)^**Ψ**^
Nonadoption, abandonment, scale-up, spread, and sustainability (NASSS) Framework	***NASSS,** Greenhalgh et al., 2017* (SR-43)^**Π**^
Health Technology Life Cycle	***HTLC,** Gutiérrez-Ibarluzea, Chiumente & Dauben, 2017* (SR-44)^**Π**^
Optimal methodology for the introduction of new orthopedic implants	***OIM-DA,** Hannan et al., 2017* (SR-45)^**Ψ**^
NASA Spaceflight Project Life Cycle	***PrLC,** NASA, 2014* (SR-46)^**Π**^
New Health Technologies – Lifecycle for integration into healthcare systems	***nHTLC4I,** Paris et al., 2017* (SR-47)^**Π**^
Life cycle of medical devices – Lifecycle approach to regulation and the importance of reporting incidents to the TGA	***TGA-MDRegLC,** Reeves & Garcia, 2014* (SR-48)^**®**^
EUnetHTA’s lifecycle approach	***EUnetHTA-MDLC,** Meyer, Brühl & Omstad, 2018* (SR-49)^**Π**^
The Device Development Process	***FDA-MDRegLC,** FDA, 2018* (SR-50)^**®**^
Lifecycle of a medical device	***Swissmedic-MDRegLC,** Swissmedic, 2019* (SR-51)^**®**^

Similar abbreviations indicate a similar name or focus: DA, decision algorithm; DOI, diffusion of innovations; EAES, European Association for Endoscopic Surgery; EIM, endoscopic innovation management; EUnetHTA, European network for Health Technology Assessment; FDA, Food and Drug Administration; HCanada, Health Canada; IC, innovation cycle; IDEAL, idea, development, exploration, assessment, and long-term study; 4S-IEE, 4-stage iterative economic evaluation; IRM, integrated risk management; IRP, innovation routinization process; MD, medical device; MDDP, medical device development process; MDRegLC, medical device regulatory lifecycle; NASA, National Aeronautics and Space Administration; nHTLC4I, new health technology life cycle for innovation; NPD, new product development; OIM, orthopedic innovation management; PLC, product life cycle; RE-AIM, reach, efficacy, adoption, implementation, maintenance; SaMDDP, software as a medical device development process; SG, stage-gate; TALC, technology adoption lifecycle; TGA, Therapeutic Goods Administration; TLC, Technology lifecycle; USVP, University Spin-off Venturing Process; VA, Veteran Administration.

**Symbols key:** **™**, Trade and Industry; ^**Π**^, Policymaking; ^**Ψ**^, Healthcare; ^**®**^, Regulation.

The models were characterized across seven dimensions related to the types of device, model, lifecycle, evaluation, and data; date of first appearance; and QA scores, which are summarized in Table 2, whilst Table 3 summarizes and compares their key features, including purpose, primary intended audience, lifecycle characterization, outputs, timing, and scope (Supplementary Material 10).

**Table 2.** Summary of model and study (reference text) characteristics

Summary of model characteristics					
Device type	HC	Policy	Reg	T&I	Total
Industry				2	2
Business				2	2
Product				7	7
Technology		3		2	5
Health technology	1	5	1		7
Medical device	4	1	7	2	14
Innovation	4	7		4	15

Lifecycle type	HC	Policy	Reg	T&I	Total
A logic used to explain a causal relationship in a variance theory		3		4	7
A category of concepts that refer to actions of individuals or organizations	7	5	8	7	27
A sequence of events that describe how things change over time	1	6		6	13
Combines both a sequence of events that describe how things change over time and a category of concepts that refer to actions of individuals or organizations	1	2		2	5

Evaluation type	HC	Policy	Reg	T&I	Total
Descriptive	1	8	4	2	15
Exploratory and/or Explanatory		3		3	6
Predictive		1		4	5
Prescriptive	8	4	4	10	26

Model type	HC	Policy	Reg	T&I	Total
Framework	4	5	7	6	22
Model	5	8	1	13	27
Theory		3			3

Data type	HC	Policy	Reg	T&I	Total
Qualitative	8	10	8	10	36
Quantitative		3		7	10
Mixed	1	3		2	6

First appearance of model	HC	Policy	Reg	T&I	Total
1930–1939				1	1
1960–1969		1		2	3
1970–1979		1			1
1980–1989		1		5	6
1990–1999	1	3	2	3	9
2000–2009	3	2	2	4	11
2010–2019	5	8	4	4	21

QA score	HC	Policy	Reg	T&I	Total
0	4	9	7	4	24
1	3	4	1	6	14
2	2	1		9	12
3		2			2
Grand total	9	16	8	19	52

Summary of reference study characteristics					
Publication period	HC	Policy	Reg	T&I	Total
1960–1969		1		3	4
1980–1989		2		5	7
1990–1999	1	3	1	2	7
2000–2009	2	2	2	4	10
2010–2019	6	8	5	5	24

Geographical region	HC	Policy	Reg	T&I	Total
Americas	2	8	4	15	29
Australia	1		1		2
Europe	3	5	1	3	12
International	3	3	2	1	9

Publication type	HC	Policy	Reg	T&I	Total
Journal article	8	9		12	29
Academic publication		1			1
Conference/Presentation		2	1	2	5
Regulatory guidance			2		2
WHO Guidance		1	2		3
Book		3	1	3	7
Magazine				1	1
Online resource	1		1		2
Web site			1	1	2

Funding/support or Conflicts (COI) declared	HC	Policy	Reg	T&I	Total
Funding/support declared	5	9	4	6	24
Personal interests stated				2	2
Writing support acknowledged		1			1
Disclaimer			1		1
Declared no COI	2	1			3
None declared	2	5	3	10	20
Uncertain				1	1
Total	9	16	8	19	52

COI, conflict of interest; HC, healthcare; NPD, new product development; Policy, policymaking; QA, quality appraisal; Reg, regulation; T&I, trade and industry; WHO, World Health Organization.

**Table 3.** Summary of findings table

Summary of findings					
Purpose	HC	Policy	Reg	T&I	Total
Describe the lifecycle		3		4	7
Explore Influencing or impacted factors		3		3	6
Identify lifecycle stage	2			1	3
Determine readiness to progress	1	2		2	5
Define actions or activities	6	8	8	9	31

Audience	HC	Policy	Reg	T&I	Total
Trade and Industry		4	3	19	26
Policymakers	1	12	2		15
Regulators	1				1
Healthcare	7	2	1		10
General public			4		4
Totals for audience	9	18	10	19	56

Characterized in terms of …	HC	Policy	Reg	T&I	Total
Development (pattern)				2	2
Development (stages)	8	7	8	10	33
Adoption (pattern)		9			9
Sales (pattern)				7	7
Health technology management activities	1				1

Outputs (Detailing …)	HC	Policy	Reg	T&I	Total
What happens	1	2	1		4
Influencing factors		3		8	11
“How to”	1	2		9	12
Process/outcomes evaluation methods or activities	7	7		2	16
Regulation		2	7		9

Timing	HC	Policy	Reg	T&I	Total
Lifecycle evaluation at a single point in time		2	1	7	10
Separate evaluation of different phases at a single timepoint	1	5		6	12
Repeated evaluation at each lifecycle stage	8	9	7	6	30

Scope – Lifecycle	HC	Policy	Reg	T&I	Total
Provision			1	19	20
Acquisition	1	2			3
Utilization		1			1
Provision and Acquisition		4			4
Provision and Utilization			6		6
Acquisition and Utilization	2	3			5
Provision and Acquisition and Utilization	6	6	1		13

Scope – Devices	HC	Policy	Reg	T&I	Total
New	8	14		17	39
Established	1			1	2
All		2	8	1	11

Model archetypes	HC	Policy	Reg	T&I	Total
NPD			1	10	11
Diffusion	1	7		9	17
Outcomes evaluation	6	5			11
Hybrid	2	4	7		13
Total	9	16	8	19	52

HC, healthcare; NPD, new product development; Policy, policymaking; Reg, regulation; T&I, trade and Industry.

### What is Meant by “Medical Device Lifecycle”?

In this section, we describe the different *forms*, *scopes*, and *types of process* used to represent the lifecycle of a medical device. The most common lifecycle *form* used is a linear series of stages (though iteration may occur) moving from the idea, through its development, growth, maturity, decline, to end-of-life (or a subset of these). A second form is a cycle of innovation, with each original invention being incrementally refined, which means that, for these models, the lifecycle encompasses several generations of a device. A third form is a pattern describing how a single lifecycle phase changes over time (e.g., sales or adoption), therefore, these models focus on how the parameter is affected rather than the effects the product has on patients/end-users. In a sense, these are not actually device lifecycles, but they are, nevertheless, often what is meant by a device’s lifecycle. Another set of models describe the lifecycle as movement through various levels in a system, and are, therefore, multi-level evaluations. For example, where a novel idea is developed into a technology, which becomes a component in a device, that is itself incorporated into a system (e.g., software developed for a switch, used in a mobile arm, which becomes a component in a robotic assistant).

**Figure 1.**
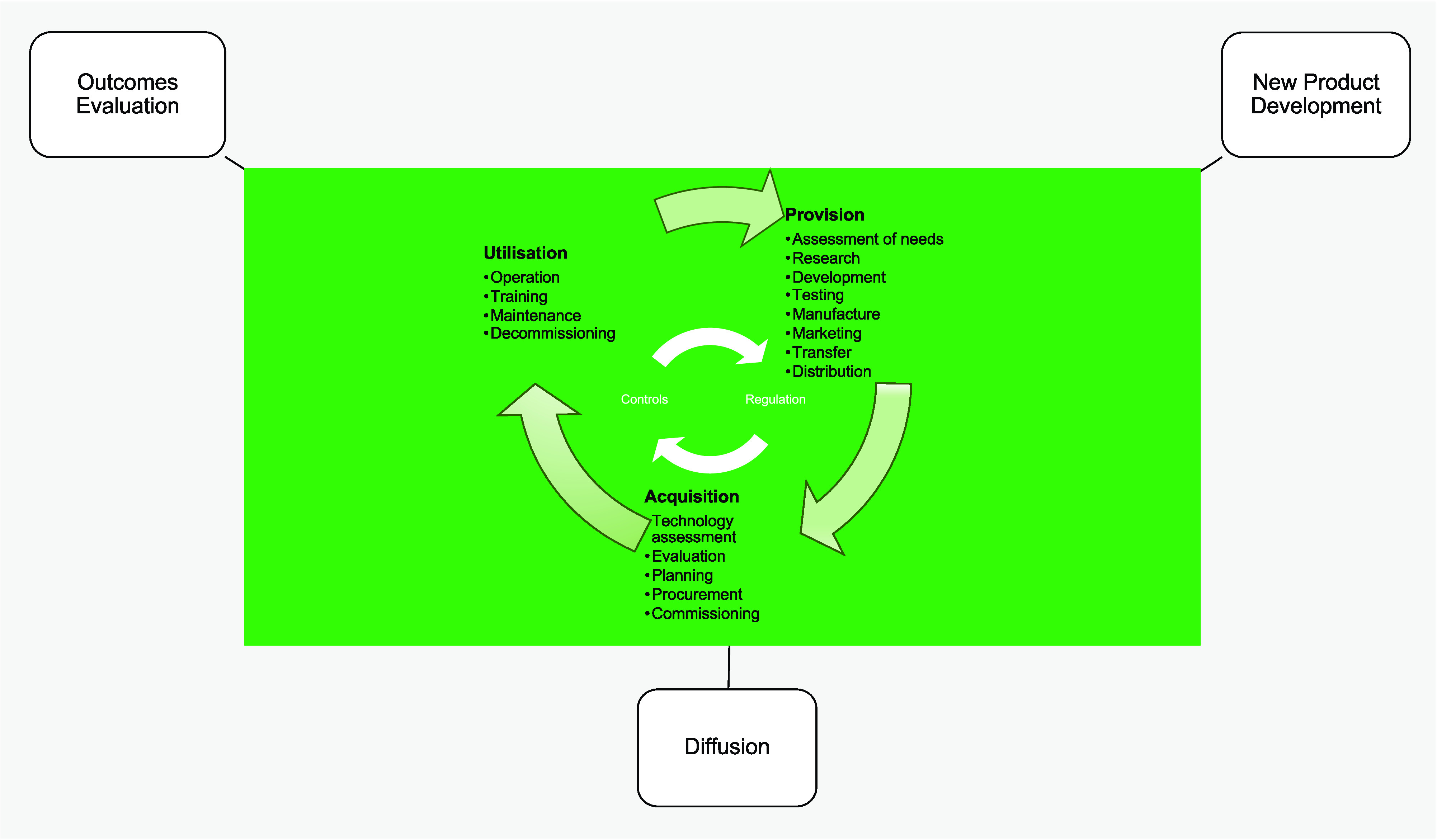
Model archetypes associated with the Healthcare Technology Life Cycle (adapted from WHO, SR-20).

To compare models’ *scope,* we utilized the Health Care Technology Lifecycle (HCTLC) model (the green part in Figure 1) from the World Health Organization (WHO) (SR-20). It illustrates the phases of the medical device lifecycle in terms of the activities/processes required across its lifespan. This includes 17 specific stages of activity grouped into three phases – Provision, Acquisition, and Utilization. Regulation may be considered as a separate process, but in this model it is considered to be an integral part, where regulatory compliance is necessary for the device to transition to the next stage. The focus of interest during the provision stage is primarily on the new product development process, whilst the acquisition phase focuses primarily on adoption or sales (i.e., diffusion). During utilization, the main focus of the models is on evaluating outcomes. However, whilst certain models cover all of these phases and stages, generally, most cover only some. In fact, Table 3 shows that the scope of the lifecycle models varied considerably across perspectives. For example, the T&I models cover only the provision stages, and approximately half of the Policy models do not include the utilization stage, which means that these evaluation models do not examine (to any great extent) what happens after a device enters the healthcare system.

To compare the *types of process* embodied in the models, we utilized Van de Ven’s process definitions – “(i) a logic used to explain a causal relationship in a variance theory, (ii) a category of concepts that refer to actions of individuals or organizations, and (iii) a sequence of events that describe how things change over time” (32, p. 169). We found that frequently the models encompassed both the second and third of these definitions. Nevertheless, approximately half of the models predominantly used a process definition that referred to actors’ actions, a quarter referred to a sequence of events, whilst five used both. The majority of these were stage models, that is they described the lifecycle as distinct stages rather than as a continuous distribution of a particular variable. This contrasts with the seven applying the first definition, exploring causal relationships, all of which characterized the lifecycle quantitatively, and none of which focused on evaluating patient/end-user outcomes.

### What is Meant by “Lifecycle Evaluation”?

As shown in Tables 2 and 3, lifecycle evaluation differs in terms of *what* gets evaluated, *when*, *how*, and *why.* Most commonly, *what* is evaluated are the factors that are used to characterize the lifecycle, indicators of lifecycle progression, factors influencing the lifecycle trajectory or that are affected by it, and/or the outcomes from deploying a technology. However, relatively few of the models, and none of those from the T&I perspective, focus primarily on evaluating patient/end-user outcomes.

An evaluation may be a once-off event encompassing the entire lifecycle in a single evaluation (e.g., using empirical data to describe the sales/adoption pattern over time or by evaluating different lifecycle stages at a single point in time, either retrospectively using existing data or prospectively using modeling techniques) or it may be a lifecycle evaluation by virtue of the fact that the evaluation is repeated at several time points across the life of a technology. It is this latter type that is generally considered to be a “lifecycle approach” by the HTA community (33). However, 22 of the models included in this synthesis did not take this approach.

*How* the lifecycle is evaluated depends on the particular purpose of a given model. It may be performed using quantitative, qualitative, or mixed types of data, to conduct a descriptive, explanatory, predictive, or prescriptive evaluation. Table 3 shows that the most frequent purpose of evaluation is to define particular actions or activities that need to be undertaken at the different lifecycle stages, only 15 of which describe approaches to evaluating end-user or patient outcomes.

The main reason for adopting a lifecycle evaluation approach is to address a need for information. The models are used for gathering, generating, or conveying information/evidence required for making the best decisions to achieve the overarching goal. The purposes listed in Table 3 are simply intermediate purposes, whilst the overarching goal (and the corresponding decision) varies across perspectives. The primary goal for HC is to provide safe, effective, and efficient healthcare, therefore, its decisions relate to the adoption of clinically effective technologies. Whilst for T&I the goal is to achieve commercial success and generate profit. To do this it must decide on the appropriate resources to invest and the timing of investment, usually in new product development and/or marketing. Policymakers often have dual policy goals – to promote trade and industry and to ensure the provision of high-quality and cost-effective healthcare. Thus, their decisions depend on the policy goal, but generally relate to market regulation or resource allocation. Sometimes, however, these goals are conflicting, because easing access to healthcare markets for trade and industry usually means reducing the quality and quantity of evidence required for market access, which reduces the evidence available for evaluating health technologies for their cost-effectiveness. “Tension can arise between these objectives. Expediency must be balanced against adequate rigor, affordability with access. Aligning regulatory objectives with broader economic and industrial policy (e.g., to promote innovation, employment, growth, export and trade) may result in tension with goals of managing costs. In addition, each objective will be prioritised differently by stakeholder groups, adding a political dimension to the process.” (SR-47, p. 118).

**Figure 2.**
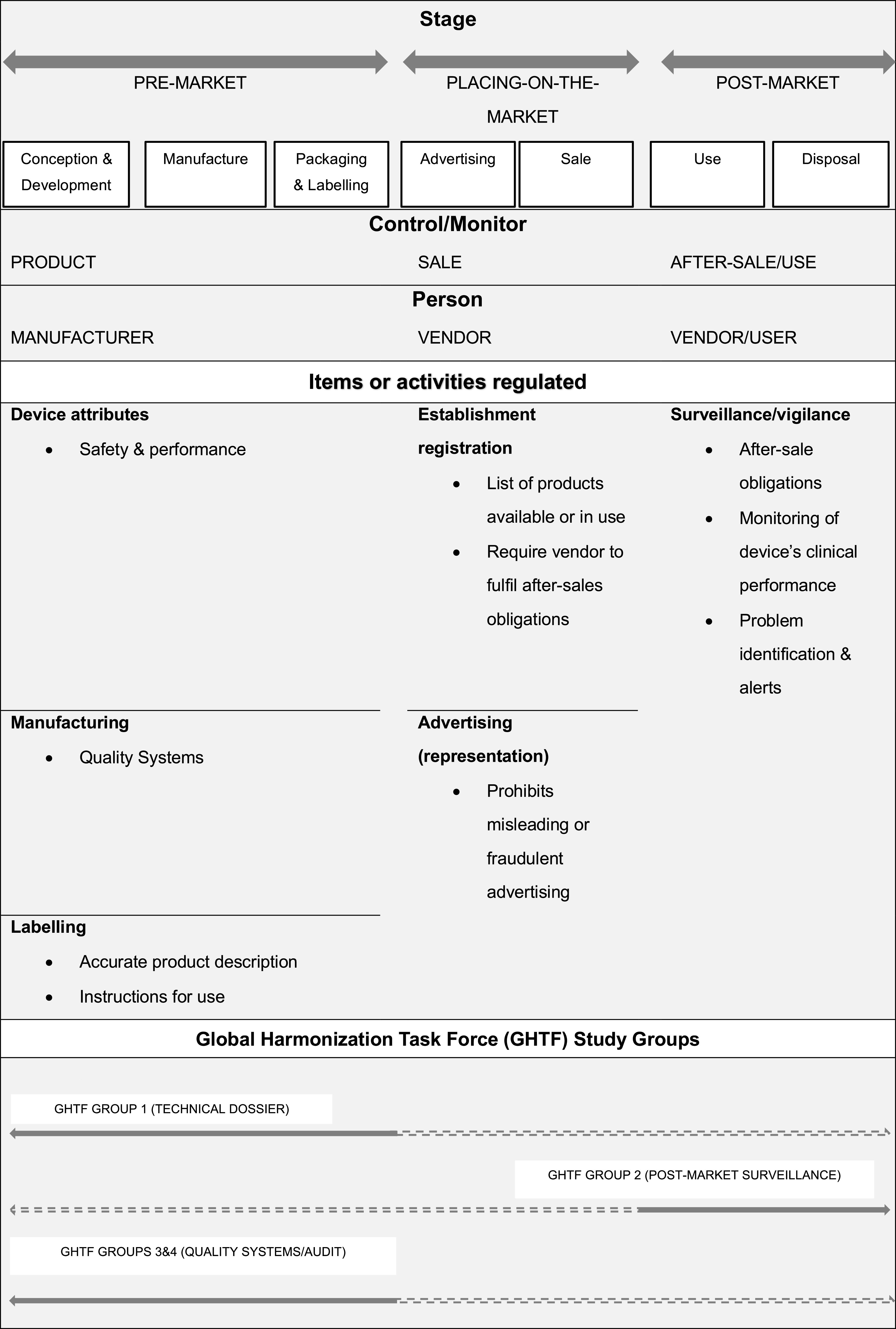
Regulation across the medical device lifecycle (adapted from WHO, SR-20).

The explicit goal for those from the regulatory perspective is not, as is frequently assumed, to ensure that medical devices entering the market are safe and effective (SR-35), but rather that the regulatory requirements of their particular jurisdiction are met. “Pre-market control is performed on the device to ensure that the ***product*** to be placed on-market complies with regulatory requirements.” (SR-20, p. 20, bold in original) Therefore, the safety and effectiveness of medical devices at market entry is dependent on the specific legal requirements in a given jurisdiction. Regulatory models differ, but for the most part regulators endorse the guidelines of the Global Harmonization Task Force and follow the approaches illustrated in Figure 2 suggested by the WHO (SR-20).

Medical device regulators are responsible for deciding whether a health technology may be legally marketed in their jurisdiction. In some jurisdictions, this *sometimes* involves a market authorization process, where the products can only be placed on the market if authorized to do so by the regulators (e.g., in the US and Canada) usually on the basis of evidence of safety and effectiveness gleaned from the literature or clinical investigations. “… the premarket notification, or 510(k), process is a classification process, whereas PMA [Pre-market Approval] is a determination of safety and effectiveness that leads to approval. … For the 510(k) process, devices are *cleared* for marketing, not approved, and devices may not be marketed as ‘approved by FDA’” (SR-23, p.12, emphasis in original). In other jurisdictions (e.g., Australia, European Union (EU), Switzerland) market authorization is not required, instead manufacturers of high-risk devices must apply for an assessment of their conformity with the legal requirements by an external body, a conformity assessment body (CAS) (SR-51). If the CAS deems them to be compliant it authorizes them to affix a marking (e.g., the CE marking in the EU) on their device, a legal requirement for marketing the device that indicates it is legally compliant. This is not the same as market authorization, “Unlike medicinal products, medical devices do not undergo an official authorisation procedure. For these devices, Switzerland follows what is specified for the European Union (EU) system of compliance assessment and certification, based on bilateral agreements. Compliance with internationally valid norms is evaluated by private entities” (SR-51).

All regulatory models include a requirement for an evaluation of clinical data for all but the lowest-risk devices, but not necessarily on the same device, nor must the clinical data include a randomized controlled trial (RCT). “What consumer protections are required to bring new products to market? … FDA is required to provide the least burdensome path to market … This means that the trials should be parsimonious in size, focused in objectives, and not cumbersome in execution. If the questions can be answered in a post-marketing period then they should not be required before marketing” (9, slides 5,14). Consequently, this regulatory lifecycle approach means that evidence generation is frequently deferred until the post-market phase (34).

## Discussion

The lack of evidence available at market entry is driving the push to adopt a lifecycle approach to evaluation for healthcare reimbursement decisions (SR-49). However, the differences noted in Tables 2 and 3 illustrate that, despite using the same words, people often mean very different things and have different reasons for using a lifecycle evaluation approach. Nevertheless, the concepts, as embodied in the model archetypes, have changed little over time. What has changed, as evidenced by the relatively recent emergence of the healthcare models, is the increasing pressure for earlier access to novel health technologies, resulting in increased pressure on healthcare providers and regulators.

However, the lack of evidence available at market entry/reimbursement is related to the regulators’ adoption of a lifecycle approach. “The concept of lifecycle regulation … also refers to an approach to regulation that factors the prospect of evidence generation postapproval into preapproval decision making” (34, p. 824). Therefore, adopting a lifecycle approach has meant that the regulators have accepted limited and poor-quality evidence, allowing devices to enter the market based on the expectation that more evidence will become available once the device is in use (34). However, this is frequently not the case (35–37).

Once devices are placed on the market regulators rely on post-market studies to address uncertainty regarding effectiveness and vigilance/surveillance activities to detect adverse outcomes. Yet, Rathi et al. have shown that many post-market studies are slow to be completed or not conducted at all (38). Whilst post-market surveillance is designed to detect only the most severe adverse outcomes, which are just a fraction of adverse outcomes that matter to patients (3;39).

Furthermore, clinicians and patients are not readily made aware of evidence regarding a device’s poor safety record, as demonstrated by Peters, Pellerin, and Janney, whose study showed that recalls of Class III devices in the US took more than 8 months to complete (40). Several authors have also demonstrated that although a device has been recalled it may still be used as a predicate (i.e., a substantially equivalent, legally marketed) device to support the market authorization of many subsequent devices through equivalence claims (38;41–44). Furthermore, because this evidence either never becomes available, or only becomes available years later, many people may be affected before it becomes clear that it is clinically inferior to alternative interventions (13–15;45).

Therefore, if HTA is to follow the example of the regulators’ lifecycle approach, it would mean accepting limited and inadequate evidence of safety and efficacy for reimbursement decisions with the promise that the required evidence would later become available. However, this assumes that the device is likely to be beneficial, unlikely to cause harm, and evidence will be made available (in a timely manner). Unfortunately, if manufacturers have already secured public funding for their devices, then there is very little incentive for them to generate additional evidence, which is costly and might show that their device is less safe or effective than alternatives, resulting in its market failure. However, without evidence the assumption that a device is safe and effective cannot be challenged or confirmed.

Industry may argue that high-quality evidence is difficult to develop due to inherent difficulties in conducting RCTs for medical devices (46), and that the costs are too great a burden for the small-to-medium enterprises (SMEs) that apparently predominate in the industry (47). However, why should patients bear the burden of having unproven devices used on them so that SMEs can be spared the cost of properly evaluating them?

Instead, governments could create healthcare infrastructures to support rigorous evidence generation in as efficient a manner as possible. There are schemes for “coverage with evidence development”, however, few devices are investigated in this way. Therefore, a more systematic approach is needed that enrolls all patients and all interventions into an ongoing and adaptive clinical trial comparing clinical outcomes for all treatments. This requires robust data collection and evaluation systems within healthcare, with secure individual patient and device identifiers, data linkage, and strong mechanisms of data protection. Such a system will require multi-stakeholder engagement and huge investment to develop, and is likely to be complex to operationalize. Nevertheless, its potential for improving clinical outcomes and patient safety is immense. Additionally, it would provide a means for industry to develop a robust evidence base to support applications for reimbursement and improve payers’ ability to fund the most cost-effective innovations. Therefore, future research should investigate how best to do this.

### Limitations

This is not an aggregative systematic review, so models exist that have not been included. Nevertheless, by including a large sample from a broad variety of sources and perspectives this review provides a reasonable picture of the general form and content of lifecycle models being used. Due to the broad inclusion criteria many of the included models are not intended specifically for medical devices, however, this heterogeneity facilitated a rich exploration of each discipline’s perspectives and motivations. Additionally, the methods we used, particularly the QA criteria, are not validated, therefore, future methodological research should establish best practices for conducting reviews of multi-disciplinary conceptual models.

## Conclusions

The medical device lifecycle and lifecycle evaluation have different meanings, and different purposes for different stakeholders. The lifecycle approach adopted by regulators has resulted in the deferral of evidence generation to the post-market period, resulting in a lack of evidence for reimbursement and clinical decisions. Therefore, research is needed to evaluate patient outcomes arising from early access to medical devices based on the promise of future evidence compared with evidence-based interventions. Furthermore, the HTA community could become involved in setting up rigorous systems within healthcare to systematically collect and analyze data on patient outcomes from the highest-risk devices (SR-44). Meanwhile, HTA bodies should not allow lifecycle evaluation to be used as an excuse for accepting inadequate evidence and should insist on appropriate evidence being made available for reimbursement decisions, as this may be the last defense some patients have against sub-optimal devices (1).

## Supporting information

Harkin et al. supplementary material 1 Harkin et al. supplementary material

Harkin et al. supplementary material 2 Harkin et al. supplementary material

Harkin et al. supplementary material 3 Harkin et al. supplementary material

Harkin et al. supplementary material 4 Harkin et al. supplementary material

Harkin et al. supplementary material 5 Harkin et al. supplementary material

Harkin et al. supplementary material 6 Harkin et al. supplementary material

Harkin et al. supplementary material 7 Harkin et al. supplementary material

Harkin et al. supplementary material 8 Harkin et al. supplementary material

Harkin et al. supplementary material 9 Harkin et al. supplementary material

Harkin et al. supplementary material 10 Harkin et al. supplementary material

## Data Availability

Access to the Excel database will be made available on reasonable request following completion of the PhD.

## Supplementary material

The supplementary material for this article can be found at https://doi.org/10.1017/S026646232300274X.
