# Supplementary material for: Lifecycle evaluation of medical devices: supporting or jeopardizing patient outcomes? A comparative analysis of evaluation models
Source: Int J Technol Assess Health Care. 2024 Jan 5;40(1):e2. doi: 10.1017/S026646232300274X (PMC10859834; doi:10.1017/S026646232300274X)
Supplement: Harkin et al. supplementary material 1 — Harkin et al. supplementary material [file S026646232300274Xsup001.pdf]

LIFECYCLE EVALUATION OF MEDICAL DEVICES – SUPPORTING OR JEOPARDIZING PATIENT OUTCOMES? A COMPARATIVE ANALYSIS OF EVALUATION MODELS

Authors: Kathleen Harkin, ORCID ID https://orcid.org/0000-0003-3260-9059; Jan Sorensen, ORCID ID https://orcid.org/0000-0003-0857-9267; Steve Thomas, ORCID ID https://orcid.org/0000-0001-9306-0114

Harkin_Supplemental-1_Definitions

Concept definitions used for this study

Evaluation

Evaluation is defined as '*the action of evaluating'*, whilst to evaluate is defined as to '*form an idea of the amount or value of something'* or to '*find a numerical expression or equivalent of something for a formula, function, or equation*.'(1) Synonyms for evaluation include analysis, assessment, appraisal, judgement, and consideration.(1) These synonyms would suggest that there are different degrees or depths of evaluation possible. For this review, any of these meanings for evaluation was considered to be acceptable, as they served to illuminate the different purposes for lifecycle evaluation.

Medical device

The definition of a medical device is broad and there are many types and kinds of medical device, with a variety of applications as illustrated in Figure 1. There are also multiple levels of conceptualisation possible for medical devices. They are technologies, which are products produced by manufacturers within the medical device industry. Thus, medical devices may be of a specific type or serve a particular function, whilst each is also a technology, a product, and possibly a business, or even an industry as illustrated in Figure 2. This means that lifecycle models used for evaluating any of these concepts might also be applicable to medical devices, even though not all industries, businesses, products, or technologies are medical devices. Furthermore, models applicable to specific medical device contexts, types, uses, or applications may also be applicable to medical devices more generally. In addition, lifecycle models focussing on innovation are often applicable to medical devices, with innovative devices representing another subset of all medical devices.

For this review, medical devices were taken to mean any of the more specific terms or any of the more broad conceptualisations, as depicted in Figures 1-2. This meant searching for lifecycle models derived from a variety of medical device conceptualisations, types, and uses.

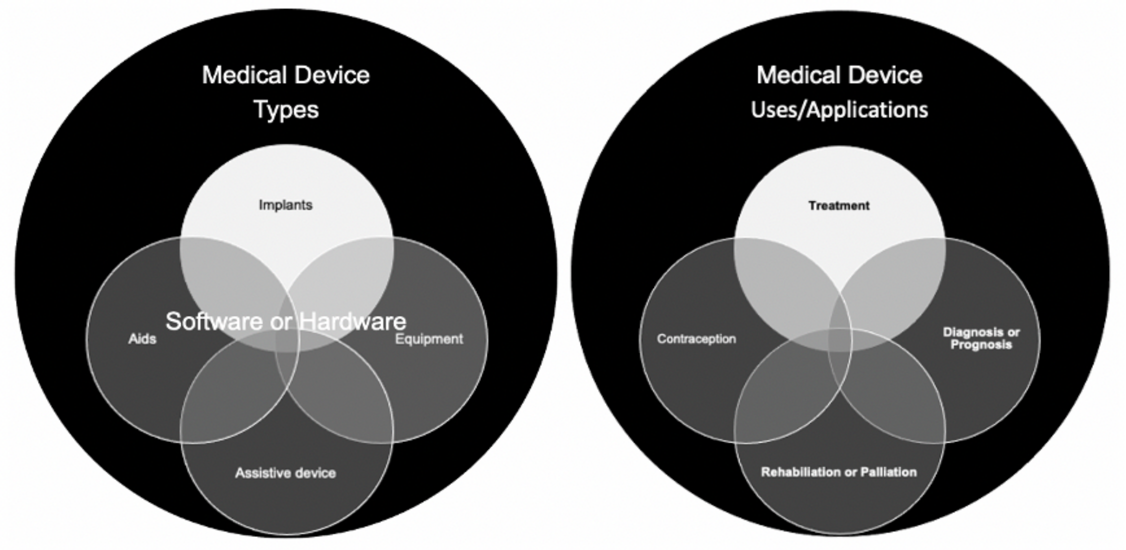

Figure 1: Multiple types, uses, and applications for a medical device

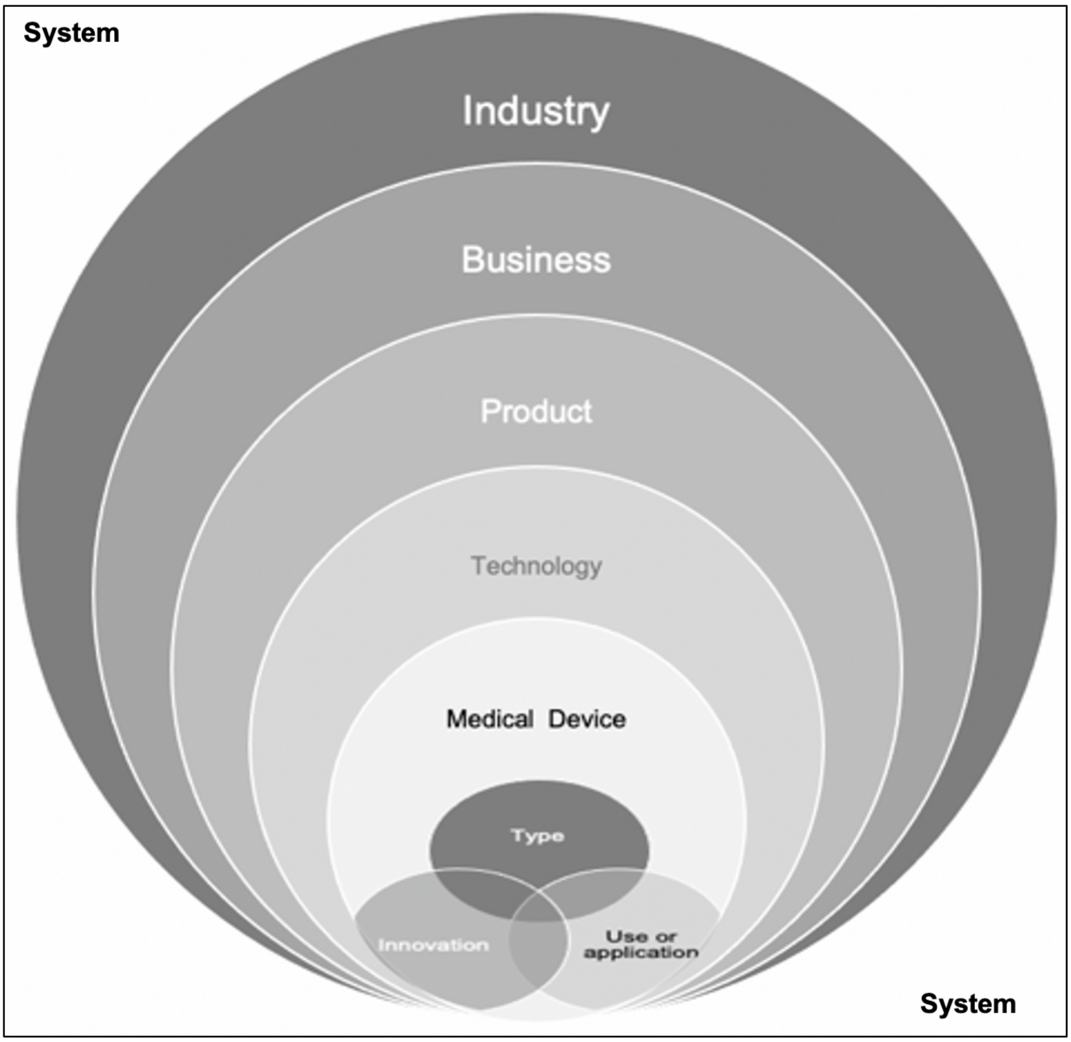

Figure 2: Conceptualising medical devices at multiple levels

Models and Frameworks

Theories, models and frameworks are variably defined, and authors often use them interchangeably.(2,3) According to Nilsen a theory is generally defined as *'a set of analytical principles or statements designed to structure our observation, understanding and explanation of the world'*.(2) It is usually composed of a set of defined variables and their relationships, including preconditions, within a given context.(2) He suggests that models are simplified versions of theories with a narrower scope of application, arguing that *'a model is descriptive, whereas a theory is explanatory as well as descriptive'*.(2) In contrast, a framework is not explanatory but rather provides a descriptive arrangement of those categories *'that are presumed to account for a phenomenon'* in the form of *'a structure, overview, outline, system or plan'*.(2)

Systematic approaches, frameworks, models, and theories create an ordered way of thinking about, and investigating, a phenomenon. They could potentially be considered to be the same concept but with differing degrees of strength of association between the variables or with differing levels of explanatory power. Therefore, this may also be considered to be a multi-level concept, as illustrated by Figure 3. Furthermore, Michie, Stralen, and West, use the term 'model' to mean "*'a hypothetical description of a complex entity or process.'*" (4), which does not limit the extent of its application.

Therefore, since this study is interested in exploring the perceptions held by different actors (i.e. what they mean by the concepts, medical device lifecycle and lifecycle evaluation) as embodied in the 'models' that they use for evaluation, all of these approaches to organizing a lifecycle evaluation of medical devices were included, and for the sake of simplicity, the term 'model' is used collectively in the article to denote any approach, framework, model or theory utilised to evaluate medical devices across their lifespan, whilst remaining cognisant of the more specific meanings.

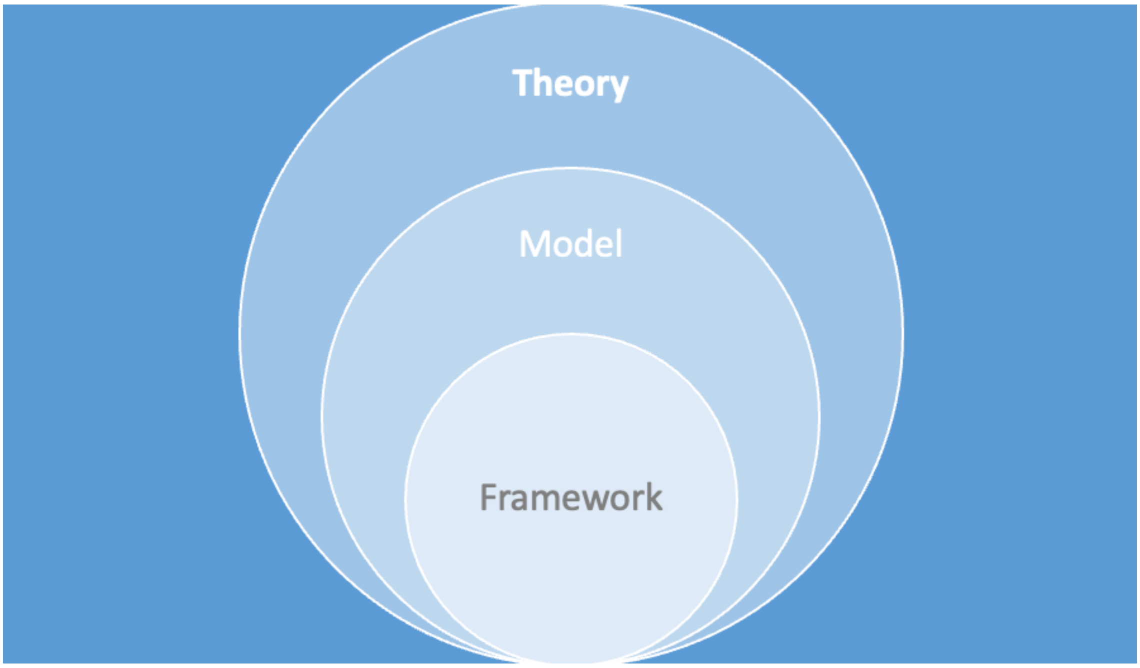

Figure 3: Multi-level conceptualisation of an evaluation 'model'

Lifecycle

Since the ‘lifecycle of a medical device’ is not explicitly defined and universally agreed, and as the purpose of this study was to explore meaning, any models that were referred to as lifecycle approaches were considered eligible for inclusion, as were any that incorporated multiple phases or processes that occur during the life of a medical device even though they may be considered incomplete ‘lifecycles’ from other perspectives. For example, the lifecycle of medical equipment as envisaged by Worm of the Tropical Health and Education Trust (2015) omits the premarket phases of a medical device.(5) Whereas, Bhuiyan’s new product development lifecycle focusses on the earlier stages and omits those phases that occur after market launch.(6)

Actors and Stakeholders

For this study an actor means someone who has an active part in a given process, whereas a stakeholder is someone who has an interest in, or is affected by, a process. Hence, all actors are stakeholders but not all stakeholders are actors. Furthermore, actors in one process may simply be stakeholders in another process. For example, manufacturers are actors within the manufacturing process, but are just stakeholders when it comes to the use or disposal of medical devices.

References

1. Waite M, Soanes C, editors. Oxford dictionary and thesaurus. 2nd ed. Oxford ; New York: Oxford University Press; 2007. 1204 p.

2. Nilsen P. Making sense of implementation theories, models and frameworks. Implement Sci. 2015 Apr 21;10(1):53.

3. Andersen O. Internationalization and market entry mode: A review of theories and conceptual frameworks. MIR Manag Int Rev. 1997;37:27–42.

4. Michie S, van Stralen MM, West R. The behaviour change wheel: A new method for characterising and designing behaviour change interventions. Implement Sci IS. 2011 Apr 23;6:42.

5. Worm A. Managing the lifecycle of medical equipment [Internet]. London, England: Tropical Health and Education Trust (THET); 2015 Sep [cited 2018 Jan 9] p. 12. Available from: https://www.thet.org/wp-content/uploads/2017/08/THET_Managing_the_medical_equipment_lifecycle_LOW-RES.pdf

6. Bhuiyan N. A framework for successful new product development. J Ind Eng Manag. 2011 Dec 11;4(4):746–70.
