# Supplementary material for: Lifecycle evaluation of medical devices: supporting or jeopardizing patient outcomes? A comparative analysis of evaluation models
Source: Int J Technol Assess Health Care. 2024 Jan 5;40(1):e2. doi: 10.1017/S026646232300274X (PMC10859834; doi:10.1017/S026646232300274X)
Supplement: Harkin et al. supplementary material 2 — Harkin et al. supplementary material [file S026646232300274Xsup002.pdf]

LIFECYCLE EVALUATION OF MEDICAL DEVICES – SUPPORTING OR JEOPARDIZING PATIENT OUTCOMES? A COMPARATIVE ANALYSIS OF EVALUATION MODELS

Authors: Kathleen Harkin, ORCID ID https://orcid.org/0000-0003-3260-9059; Jan Sorensen, ORCID ID https://orcid.org/0000-0003-0857-9267; Steve Thomas, ORCID ID https://orcid.org/0000-0001-9306-0114

Harkin_Supplemental-2_Search strategies

Search strategies

Systematic search strategies were developed in PubMed for scoping the topic, and for determining the best approach to searching. The systematic scoping searches were conducted between July 2018 and June 2019 inclusive, whilst pragmatic searching began in Jan 2018 and continued until May 2022, iterating with analysis.

Pragmatic searching

The approach to searching was iterative, so that where a set of terms in a particular resource (e.g. Google, JSTOR, etc.) provided particularly promising results, further searches were conducted in those resources using similar or different terms or following citations or links to similar articles, and citations or links from those to still others, essentially following a thread of connections. In this way multiple searches were conducted. However, systematic recording of these searches was difficult as they didn't follow the typical systematic search approach, recording of these searches (though attempted) was, therefore, abandoned. Table 1 provides an example of the searching approach adopted for pragmatic searching using Google.

Table 1: Pragmatic searching example

**Search Resource: Google via Firefox browser.**				
**Search term**	**Number of hits (About*)**	**Academic paper(s) identified – Author, year (Reference)**	**Link or other relevant link identified**	**Perspective**
Medical device lifecycle	5,490,000*		Lifecycle of a Medical Device / IVD - TGA	Regulator
Product lifecycle	97,900,000*	Theodore Levitt, 1965 (1)	Exploit the Product Life Cycle - Harvard Business Review	Business
Industry lifecycle	142,000,000*	Klepper, 1997 (2)		Economics
Innovation lifecycle	53,600,000*		FDA TPLC - Total Product Life Cycle - FDA	Regulator
Technology lifecycle	39,400,000*	Duretec & Becker, 2017 (3)	Technology Lifecycle Management - UNICOM Government	Government

Diffusion of innovations	1,330,000*	Rogers, (4) Greenhalgh, 2004 (5)		Policymaking

Search results were screened to identify potentially relevant texts at the time of searching. Only the first few (up to ten) pages of hits on Google were searched as Google sorts them according to their relevance (advertisements and promoted websites were ignored). Screening was done in a phased manner, first assessing if the article or webpage potentially contained a lifecycle approach or model, with potentially relevant texts being downloaded into a document archive for further screening against the inclusion criteria.

Systematic scoping searches

The following searches document some of the development of a set of systematic search strategies in the biomedical literature. Ultimately, the number of articles to screen across multiple disciplinary databases made a systematic searching approach infeasible. Consequently, it was decided to adopt a pragmatic approach to searching and to conduct a configurative review rather than an aggregative review in order to understand the essence of phenomenon rather than to quantify it.

Initial searches

Although there are many other proposed frameworks for constructing search strategies (e.g. SPIDER, SPICE, PerSPE(C)TiF, etc.),(6–9) the PICO framework was used to guide the conceptual development of the search strategy since it is considered a flexible framework for structuring focussed research questions.(10)

PICO Search Strategy 1

Table 2: PICO Search PubMed 27 July 2018

PICO 20180727 PICO
(((((((Device[Title/Abstract] OR devices)[Title/Abstract] OR (Technology[Title/Abstract] OR technologies)[Title/Abstract] OR (Intervention[Title/Abstract] OR interventions[Title/Abstract] OR interventional)[Title/Abstract] OR (Innovation[Title/Abstract] OR innovations[Title/Abstract] OR innovative)))[Title/Abstract]))) AND ((((life OR "life span" OR lifecycle))) AND ((framework or model or conceptual)))
73,431 hits in PubMed on 27th July 2018

PICO Search Strategy 2

This second PICO search strategy drew on the systematic search strategy developed by a group of researchers from the Australian Safety and Efficacy Register of New Interventional Procedures – Surgical (ASERNIP-S) for a systematic review of the literature, commissioned by the WHO, on the needs of the elderly for medical devices.(11, p.11)

Table 3: PICO search in PubMed 26 Oct 2018

PubMed 20181029 PICO
((((((((((((((((((((((((equipment and supplies[MeSH Terms]))) OR assistive devices[MeSH Terms]) OR surgical instruments[MeSH Terms]) OR device*) OR aid*) OR equipment) OR armamentarium) OR appliance*) OR instrument*) OR apparatus) OR good*) OR implement*) OR material*) OR machine*) OR prosthe*) OR implant*) OR technolog*) OR intervention*))<br>AND<br>((((((((((((((((diagnosis[MeSH Terms]) OR rehabilitation[MeSH Terms]) OR secondary prevention[MeSH Terms]) OR after treatment[MeSH Terms]) OR therapeutics[MeSH Terms]) OR diagnos*) OR therap*) OR treatment) OR prevention) OR monitoring) OR screening) OR rehabilitat*) OR alleviat*) OR procedure*) OR surgery) OR surgical)))<br>OR ((((prostheses and implants[MeSH Terms]))) OR "medical device*")))<br>AND<br>(((((((cycle, life[MeSH Terms]) OR lifespan) OR lifecycle*) OR life-cycle*) OR “life cycle”))<br>NOT<br>((animal[MeSH Terms]) NOT humans[MeSH Terms]))
8,759 hits in PubMed 26th Oct 2018 (using WHO approach to searching for meddevs)(REF)

Table 4: PICO Search in Cochrane Library 26 Oct 2018

Cochrane Search strategy 20181026<br>Search Name: Lifecycle AND Generic MedDev Search Terms<br>Date Run: 26/10/2018 21:48:19<br>Comment: Lifecycle terms and MedDev terms based on a modification of the WHO search strategy for the systematic review of needs of the ageing population	
ID	Search
#1	MeSH descriptor: [Equipment and Supplies] explode all trees
#2	MeSH descriptor: [Self-Help Devices] explode all trees
#3	MeSH descriptor: [Surgical Instruments] explode all trees
#4	device*

#5	aid*
#6	equipment
#7	armamentarium
#8	appliance*
#9	instrument*
#10	apparatus
#11	good*
#12	implement*
#13	material*
#14	machine*
#15	prosthe*
#16	implant*
#17	technolog*
#18	intervention*
#19	#1 OR #2 OR #3 OR #4 OR #5 OR #6 OR #7 OR #8 OR #9 OR #10 OR #11 OR #12 OR #13 OR #14 OR #15 OR #16 OR #17 OR #18
#20	MeSH descriptor: [Diagnosis] explode all trees
#21	MeSH descriptor: [Rehabilitation] explode all trees
#22	MeSH descriptor: [Secondary Prevention] explode all trees
#23	MeSH descriptor: [Aftercare] explode all trees
#24	MeSH descriptor: [Therapeutics] explode all trees
#25	diagnos*
#26	therap*
#27	treatment
#28	prevention
#29	monitoring
#30	screening
#31	rehabilitat*
#32	alleviat*
#33	procedur*
#34	surgery
#35	surgical
#36	#20 OR #21 OR #22 OR #23 OR #24 OR #25 OR #26 OR #27 OR #28 OR #29 OR #30 OR #31 OR #32 OR #33 OR #34 OR #35
#37	#19 AND #36
#38	MeSH descriptor: [Prostheses and Implants] explode all trees

#39	"medical device*"
#40	#38 OR #39
#41	#37 OR #40
#42	MeSH descriptor: [Life Cycle Stages] explode all trees
#43	lifespan
#44	lifecycle*
#45	life-cycle*
#46	“life cycle”
#47	#42 OR #43 OR #44 OR #45 OR #46
#48	#41 AND #47
702 hits in Cochrane 26th Oct 2018	

Table 5: PICO Search in EMBASE 26 Oct 2018

EMBASE Search strategy 20181026, based on the above search strategies, which were developed initially in PubMed (based on Lifecycle terms and MedDev terms based on a modification of the WHO search strategy for the systematic review of needs of the ageing population).	
#71	#55 AND #70
#70	#64 NOT #69
#69	'animal'/de NOT 'human'/de
#68	'animal'/de
#67	'human'/de
#66	'human'/exp
#65	#55 AND #64
#64	#57 OR #58 OR #59 OR #60
#63	#57 OR #58 OR #59 OR #60 OR #61 OR #62
#62	'life cycle stage'/exp
#61	'life cycle stage'
#60	'life cycle'
#59	lifecycle*
#58	lifespan
#57	'life cycle'/exp
#55	#51 OR #54
#54	#52 OR #53
#53	'implant'/exp
#52	'prosthesis'/exp
#51	#49 AND #50

#50	#13 OR #29
#49	#30 OR #31 OR #32 OR #33 OR #34 OR #35 OR #36 OR #37 OR #38 OR #39 OR #40 OR #41 OR #42 OR #43 OR #44 OR #45 OR #46 OR #47 OR #48
#48	medical
#47	surgical
#46	surgery
#45	procedure*
#44	alleviat*
#43	rehabilitat*
#42	screening
#41	monitoring
#40	prevention
#39	treatment
#38	therap*
#37	diagnos*
#36	'therapy'/exp
#35	'aftercare'/exp
#34	'secondary prevention'/exp
#33	'diagnostic procedure'/exp
#32	'diagnostic procedure'/exp
#31	'rehabilitation'/exp
#30	'procedures'/exp
#29	#14 OR #15 OR #16 OR #17 OR #18 OR #19 OR #20 OR #21 OR #22 OR #23 OR #24 OR #25 OR #26 OR #27 OR #28
#28	intervention*
#27	technolog*
#26	implant*
#25	prosthe*
#24	machine*
#23	material*
#22	implement*
#21	good*
#20	apparatus
#19	instrument*
#18	appliance*

#17	armamentarium
#16	equipment
#15	aid*
#14	device*
#13	#1 OR #2 OR #3 OR #4 OR #5 OR #6 OR #7 OR #8 OR #9 OR #10 OR #11 OR #12
#12	surgical NEXT/2 device*
#11	surgical NEXT/2 material*
#10	surgical NEXT/2 good*
#9	surgical NEXT/2 apparatus
#8	surgical NEXT/2 implement*
#7	surgical NEXT/2 armamentarium
#6	surgical NEXT/2 instrument*
#5	surgical NEXT/2 equipment
#4	surgical NEXT/2 applianc*
#3	surgical NEXT/2 aid*
#2	device*
#1	'medical device'/exp OR 'medical device'
34,962 hits in EMBASE 26th Oct 2018	

BeHEMoTh Search strategy

Since the PICO framework was yielding far too many hits with poor specificity, an alternative approach was tried. We used the BeHEMoTh approach, which was developed specifically as a systematic approach to searching for theory, to attempt to increase both the sensitivity and specificity of the search results.(12) The research question is built up around three concepts - the '**Be**haviour of interest', the **H**ealth context, and **Mo**dels or **Th**eories. The E in BeHEMoTh refers to **E**xclusion of non-theoretical models (e.g. models of care). We conceptualised these as illustrated in the table below.

BeHEMoTh element	Conceptualised as:	Search terms
**Be**haviour of interest'	**Medical devices**	((((((((((((((((((((((((((((((((((((equipment and supplies[MeSH Terms]))) OR assistive devices[MeSH Terms]) OR surgical instruments[MeSH Terms]) OR device*) OR aid*) OR equipment) OR armamentarium) OR appliance*) OR instrument*) OR apparatus) OR good*) OR implement*) OR material*) OR machine*) OR prosthe*) OR implant*) OR technolog*) OR intervention*))))) AND

		(((((((((((((((((diagnosis[MeSH Terms]) OR rehabilitation[MeSH Terms]) OR secondary prevention[MeSH Terms]) OR after treatment[MeSH Terms]) OR therapeutics[MeSH Terms]) OR diagnos*) OR therap*) OR treatment) OR prevention) OR monitoring) OR screening) OR rehabilitat*) OR alleviat*) OR procedure*) OR surgery) OR surgical)))))) OR (("medical device*") OR ((prostheses and implants[MeSH Terms])))))
**AND**		
**H**ealth context	**Lifecycle**	((((((cycle, life[MeSH Terms]) OR lifespan) OR lifecycle*) OR life-cycle*) OR "life cycle"))))
**NOT**		
**E**xclusions	**Other types of model**	((((("Models, Statistical"[Mesh]) OR "Models, Animal"[Mesh])) OR "Fractals"[Mesh]) OR "Models, Nursing"[Mesh])))
**AND**		
**Mo**dels or **Th**eories	**Conceptual models**	((((theor*) OR framework*) OR model*) OR concept*)

The results of these searches are provided below. However, this approach was developed for use within a single disciplinary paradigm, and we found it less useful across multiple disciplines and research traditions since the number of results this yielded from the biomedical databases alone was still too large to be a feasible approach for searching within all the relevant bibliographic databases for the multiple other disciplines involved across the life of a medical device. Therefore, the pragmatic approach to searching described above was adopted.

Table 6: BeHEMoTH Search in PubMed 30 Jan 2019

PubMed Search strategy 20190130 BeHEMoTh
((((((((((((((((((((((((((((((((((((equipment and supplies[MeSH Terms]))) OR assistive devices[MeSH Terms]) OR surgical instruments[MeSH Terms]) OR device*) OR aid*) OR equipment) OR armamentarium) OR appliance*) OR instrument*) OR apparatus) OR good*) OR implement*) OR material*) OR machine*) OR prosthe*) OR implant*) OR technolog*) OR intervention*)))))   AND   (((((((((((((((((diagnosis[MeSH Terms]) OR rehabilitation[MeSH Terms]) OR secondary prevention[MeSH Terms]) OR after treatment[MeSH Terms]) OR therapeutics[MeSH Terms]) OR diagnos*) OR therap*) OR treatment) OR prevention)

OR monitoring) OR screening) OR rehabilitat*) OR alleviat*) OR procedure*) OR surgery) OR surgical))))))   OR (("medical device*") OR ((prostheses and implants[MeSH Terms]))))) <br>AND ((((((cycle, life[MeSH Terms]) OR lifespan) OR lifecycle*) OR life-cycle*) OR "life cycle"))))   NOT (((((("Models, Statistical"[Mesh]) OR "Models, Animal"[Mesh])) OR "Fractals"[Mesh]) OR "Models, Nursing"[Mesh])))   AND ((((theor*) OR framework*) OR model*) OR concept*)
3,825 hits in PubMed 30th January 2019

Table 7: BeHEMoTH Search in Cochrane Library 30 Jan 2019

Cochrane Search Strategy		
Search Name:	Meddev lifecycles BeHEMoTh 20190130	
Date Run:	30/01/2019 20:09:13	
Comment:	Lifecycle terms and MedDev terms based on a modification of the WHO search strategy for the systematic review of needs of the ageing population NOT E (other models) AND theory/model terms	
ID	Search	Hits
#1	MeSH descriptor: [Equipment and Supplies] explode all trees	
#2	MeSH descriptor: [Self-Help Devices] explode all trees	
#3	MeSH descriptor: [Surgical Instruments] explode all trees	
#4	device*	
#5	aid*	
#6	equipment	
#7	armamentarium	
#8	appliance*	
#9	instrument*	
#10	apparatus	
#11	good*	
#12	implement*	
#13	material*	
#14	machine*	
#15	prosthe*	
#16	implant*	
#17	technolog*	
#18	intervention*	

#19	#1 OR #2 OR #3 OR #4 OR #5 OR #6 OR #7 OR #8 OR #9 OR #10 OR #11 OR #12 OR #13 OR #14 OR #15 OR #16 OR #17 OR #18
#20	MeSH descriptor: [Diagnosis] explode all trees
#21	MeSH descriptor: [Rehabilitation] explode all trees
#22	MeSH descriptor: [Secondary Prevention] explode all trees
#23	MeSH descriptor: [Aftercare] explode all trees
#24	MeSH descriptor: [Therapeutics] explode all trees
#25	diagnos*
#26	therap*
#27	treatment
#28	prevention
#29	monitoring
#30	screening
#31	rehabilitat*
#32	alleviat*
#33	procedur*
#34	surgery
#35	surgical
#36	#20 OR #21 OR #22 OR #23 OR #24 OR #25 OR #26 OR #27 OR #28 OR #29 OR #30 OR #31 OR #32 OR #33 OR #34 OR #35
#37	#19 AND #36
#38	MeSH descriptor: [Prostheses and Implants] explode all trees
#39	"medical device*"
#40	#38 OR #39
#41	#37 OR #40
#42	MeSH descriptor: [Life Cycle Stages] explode all trees
#43	lifespan
#44	lifecycle*
#45	life-cycle*
#46	‘life cycle’
#47	#42 OR #43 OR #44 OR #45 OR #46
#48	#41 AND #47
#49	MeSH descriptor: [Models, Statistical] explode all trees
#50	MeSH descriptor: [Models, Animal] explode all trees
#51	MeSH descriptor: [Models, Nursing] explode all trees
#52	MeSH descriptor: [Fractals] explode all trees

#53	#49 OR #50 OR # 52 OR #52
#54	#48 NOT #53
#55	theor*
#56	framework*
#57	concept*
#58	model*
#59	#55 OR #56 OR #57 OR #58
#60	#54 AND #59
194 hits in Cochrane 30th January 2019	

Table 8: BeHEMoTH Search in EMBASE 30 Jan 2019

EMBASE Search strategy 20190130 BeHEMoTh	
#1	(('devices'/exp OR device* OR aid* OR equipment OR armamentarium OR appliance* OR instrument* OR apparatus OR good* OR implement* OR material* OR machine* OR prosthe* OR implant* OR technolog* OR intervention*) AND ('procedures'/exp OR 'health care delivery'/exp OR diagnos* OR therap* OR treatment OR prevention OR monitoring OR screening OR rehabilitat* OR alleviat* OR procedure* OR surgery OR surgical) OR 'medical device'/exp OR (medical AND device*)) AND ('theoretical model'/exp OR 'conceptual framework'/exp OR framework* OR model* OR theor* OR concept*) AND ('life cycle'/exp OR 'life cycle assessment'/exp OR lifespan OR lifecycle* OR 'life cycle*')
#2	disease model'/exp OR 'nursing theory'/exp OR 'statistical model'/exp OR 'animal model'/exp
#1 NOT #2	((('devices'/exp OR device* OR aid* OR equipment OR armamentarium OR appliance* OR instrument* OR apparatus OR good* OR implement* OR material* OR machine* OR prosthe* OR implant* OR technolog* OR intervention*) AND ('procedures'/exp OR 'health care delivery'/exp OR diagnos* OR therap* OR treatment OR prevention OR monitoring OR screening OR rehabilitat* OR alleviat* OR procedure* OR surgery OR surgical) OR 'medical device'/exp OR (medical AND device*)) AND ('theoretical model'/exp OR 'conceptual framework'/exp OR framework* OR model* OR theor* OR concept*) AND ('life cycle'/exp OR 'life cycle assessment'/exp OR lifespan OR lifecycle* OR 'life cycle*')) NOT ('disease model'/exp OR 'nursing theory'/exp OR 'statistical model'/exp OR 'animal model'/exp)

10,966 hits in EMBASE 30th January 2019

Summary of systematic searches

Table 9: Summary of search results

	PICO Search Strategy 1	PICO Search strategy 2	BeHEMoTh Search strategy	
**Database**	#Hits 27/07/2018	#Hits 26/10/2018	#Hits 30/01/2019	#Hits 30/06/2019
**PubMed**	73,431	8,759	3,825	3914
**EMBASE**	-	34,962	10,966	11,458
**Cochrane Library**	-	702	194	225
**Total # hits**	73,431	44,423	14,985	15,597

References

1. Levitt T. Exploit the Product Life Cycle. Harv Bus Rev. 1965 Nov 1;43(6):81–94.
2. Klepper S. Industry life cycles. Ind Corp Change. 1997 Jan 1;6(1):145–82.
3. Duretec K, Becker C. Format technology lifecycle analysis. J Assoc Inf Sci Technol. 2017 Oct 1;68(10):2484–500.
4. Rogers EM. Diffusion of innovations. 3rd ed. New York : London: Free Press ; Collier Macmillan; 1983. 453 p.
5. Greenhalgh T, Robert G, Macfarlane F, Bate P, Kyriakidou O. Diffusion of innovations in service organizations: Systematic review and recommendations. Milbank Q. 2004;82(4):581–629.
6. Cooke A, Smith D, Booth A. Beyond PICO: the SPIDER tool for qualitative evidence synthesis. Qual Health Res. 2012 Oct;22(10):1435–43.
7. Booth A. Clear and present questions: formulating questions for evidence based practice. Cleyle S, editor. Libr Hi Tech. 2006 Jan 1;24(3):355–68.
8. Booth A, Noyes J, Flemming K, Moore G, Tunçalp Ö, Shakibazadeh E. Formulating questions to explore complex interventions within qualitative evidence synthesis. BMJ Glob Health. 2019 Jan;4(Suppl 1):e001107.
9. Flemming K, Noyes J. Qualitative Evidence Synthesis: Where are we at? Int J Qual Methods. 2021 Jan 1;20:1609406921993276.
10. Glasziou PP, Chris DM, Salisbury J. Evidence-Based Practice Workbook. 2nd ed. Malden, Mass. Oxford: BMJ Books; 2007. 208 p.
11. Australian Safety and Efficacy Register of New Interventional Procedures – Surgical (ASERNIP-S). Systematic review of needs for medical devices for ageing populations [Internet]. Switzerland: World Health Organization (WHO); 2015 [cited 2018 Sep 21] p. 116. Available from: https://www.who.int/medical_devices/systematic_Review_needs.pdf
12. Booth A, Carroll C. Systematic searching for theory to inform systematic reviews: Is it feasible? Is it desirable? Health Inf Libr J. 2015;32(3):220–35.
