# Supplementary material for: Lifecycle evaluation of medical devices: supporting or jeopardizing patient outcomes? A comparative analysis of evaluation models
Source: Int J Technol Assess Health Care. 2024 Jan 5;40(1):e2. doi: 10.1017/S026646232300274X (PMC10859834; doi:10.1017/S026646232300274X)
Supplement: Harkin et al. supplementary material 3 — Harkin et al. supplementary material [file S026646232300274Xsup003.pdf]

LIFECYCLE EVALUATION OF MEDICAL DEVICES – SUPPORTING OR JEOPARDIZING PATIENT OUTCOMES? A COMPARATIVE ANALYSIS OF EVALUATION MODELS

Authors: Kathleen Harkin, ORCID ID https://orcid.org/0000-0003-3260-9059; Jan Sorensen, ORCID ID https://orcid.org/0000-0003-0857-9267; Steve Thomas, ORCID ID https://orcid.org/0000-0001-9306-0114

Harkin_Supplemental-3_Inclusion & Exclusion Criteria

Study inclusion and exclusion criteria using the PICOTS framework

Inclusion and exclusion criteria were developed based on the PICOTS framework, as shown in Table 1, since it is simple and is considered a flexible framework suitable for developing focussed research questions for both quantitative and qualitative study designs.(1, p.24)

Table 1: Inclusion and exclusion criteria for selecting models

PICOTS	*Inclusion Criteria*	*Exclusion Criteria*
**P**opulation/<br>**P**henomenon	Medical devices<br>Conceptualised as a specific type of device, or as a product, industry, innovation, therapy, technology, intervention, software or hardware	Medicines<br>(excluded because the lifecycle stages are quite different, with different evaluation problems and requirements)<br>Focus on environmental impacts
**I**ntervention/**I**nterest	Lifecycle, lifetime, lifespan, multi-stage, or multiple processes, activities or phases within a stage (i.e. it must cover more than one process, activity, or time point)	Single process or activity, or examines only a single point in time
**C**omparator/**C**ontext	Not specified<br>However, searches for models were conducted across those disciplines involved in medical device lifecycle activities, including design, biomedical engineering, manufacturing, marketing, regulation, HTA, procurement, health sciences, and quality management.	Not specified

**O**utcome/**O**utput	Model, framework, theory, or approach to the evaluation of a medical device across its lifespan	Eco-innovation models focussed on assessing innovations to improve environmental impacts
**T**imeframe	Not limited	
**S**tudy Design	All 'designs' were considered eligible, including: reports, research articles, theoretical or conceptual papers, descriptive studies, guidelines, commentaries, books, summaries of books, chapters in books or webpages.	Where there were books and articles on the same topic/results the book was omitted (due to the size), and where there were chapters of books that were relevant, the remainder of the book was excluded. Summaries of books were preferred to whole texts, particularly where the book was unavailable.

References

1. Glasziou PP, Chris DM, Salisbury J. Evidence-Based Practice Workbook. 2 edition. Malden, Mass. Oxford: BMJ Books; 2007. 208 p.
