# Supplementary material for: Lifecycle evaluation of medical devices: supporting or jeopardizing patient outcomes? A comparative analysis of evaluation models
Source: Int J Technol Assess Health Care. 2024 Jan 5;40(1):e2. doi: 10.1017/S026646232300274X (PMC10859834; doi:10.1017/S026646232300274X)

LIFECYCLE EVALUATION OF MEDICAL DEVICES – SUPPORTING OR JEOPARDIZING PATIENT OUTCOMES? A COMPARATIVE ANALYSIS OF EVALUATION MODELS

Authors: Kathleen Harkin, ORCID ID https://orcid.org/0000-0003-3260-9059; Jan Sorensen, ORCID ID https://orcid.org/0000-0003-0857-9267; Steve Thomas, ORCID ID https://orcid.org/0000-0001-9306-0114

Harkin_Supplemental-4_Screening & Selection

Screening & Selection Process

1. Initial screening of identified titles, abstracts, and certain full texts took place during the searching process to identify texts that potentially used a lifecycle model or lifecycle approach and to exclude irrelevant texts. Documents (including webpages) that appeared relevant were downloaded and saved in a Dropbox folder, which served as a ***Document Archive*** for further screening.
2. Archived documents were screened against the PICOTS inclusion and exclusion criteria (Supplemental-3). Data from selected texts were extracted into tables in Word and into an ***Excel Database***. Data extraction and analysis were concurrent with screening and selection.
3. A **selection decision algorithm** was devised (inset in the Screening & Selection Process Map) to identify the most relevant, and to choose (as far as possible) a single document to represent each model. This algorithm served as a guide rather than as a strict set of rules.
4. The final selection involved choosing between the originator article and others that extended its description, depicted or demonstrated it, described its use, or otherwise added to the analysis in order to select one text as the representative reference text. The others, though 'not selected' were not excluded, instead they served as additional reference materials.
5. Searching, screening, sampling of documents from the archive, and selection for inclusion in the excel database continued until data saturation was reached.
   Saturation was reached before the entire Document Archive had been screened, nevertheless, the remainder of the documents in the archive were also screened. On completion of screening any additional models that were found to meet the inclusion criteria were retained for triangulation of the study's findings.

Figure S4-1: Screening & Selection Process Map

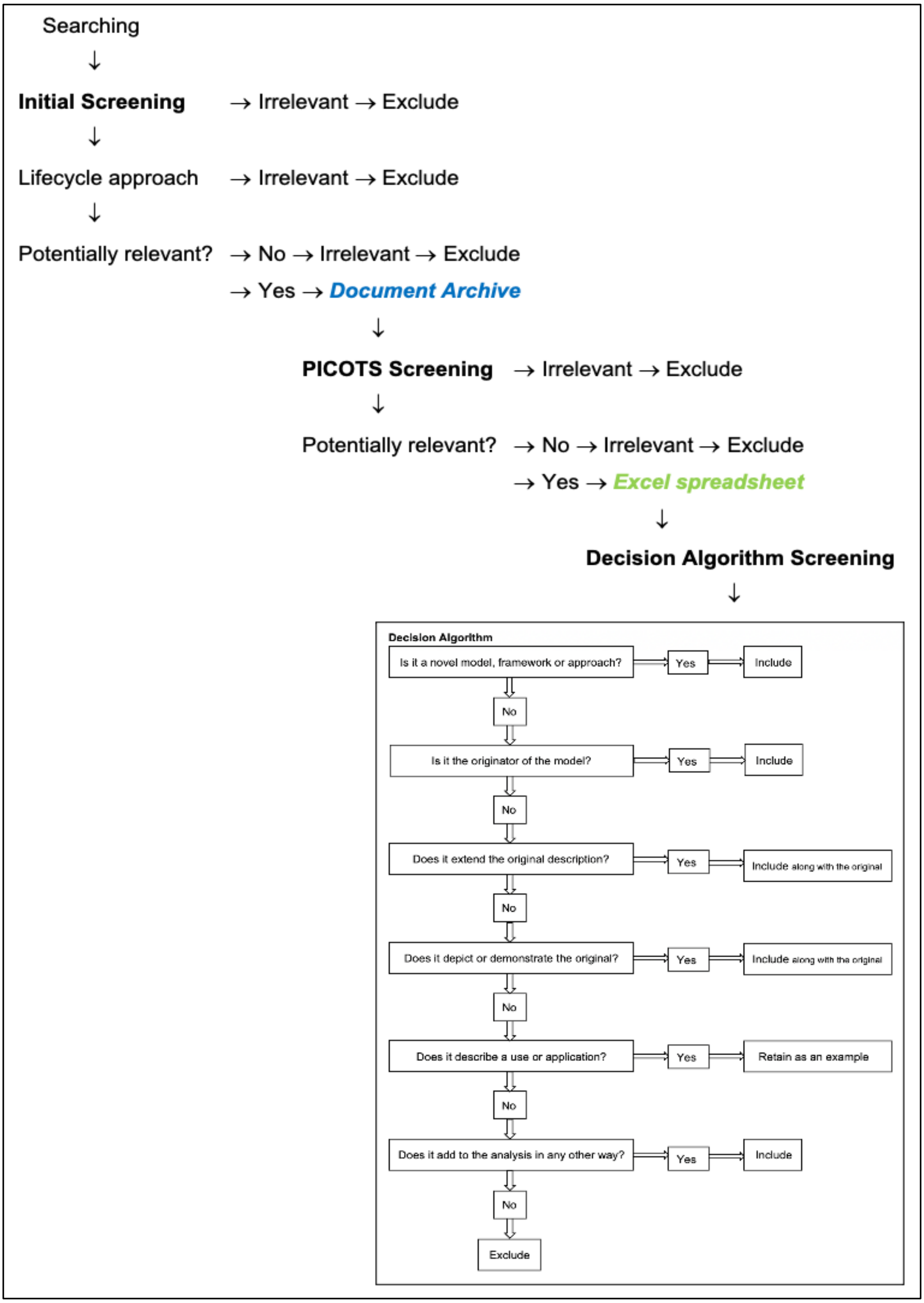

Figure S4-2: Model Screening & Selection Flowchart

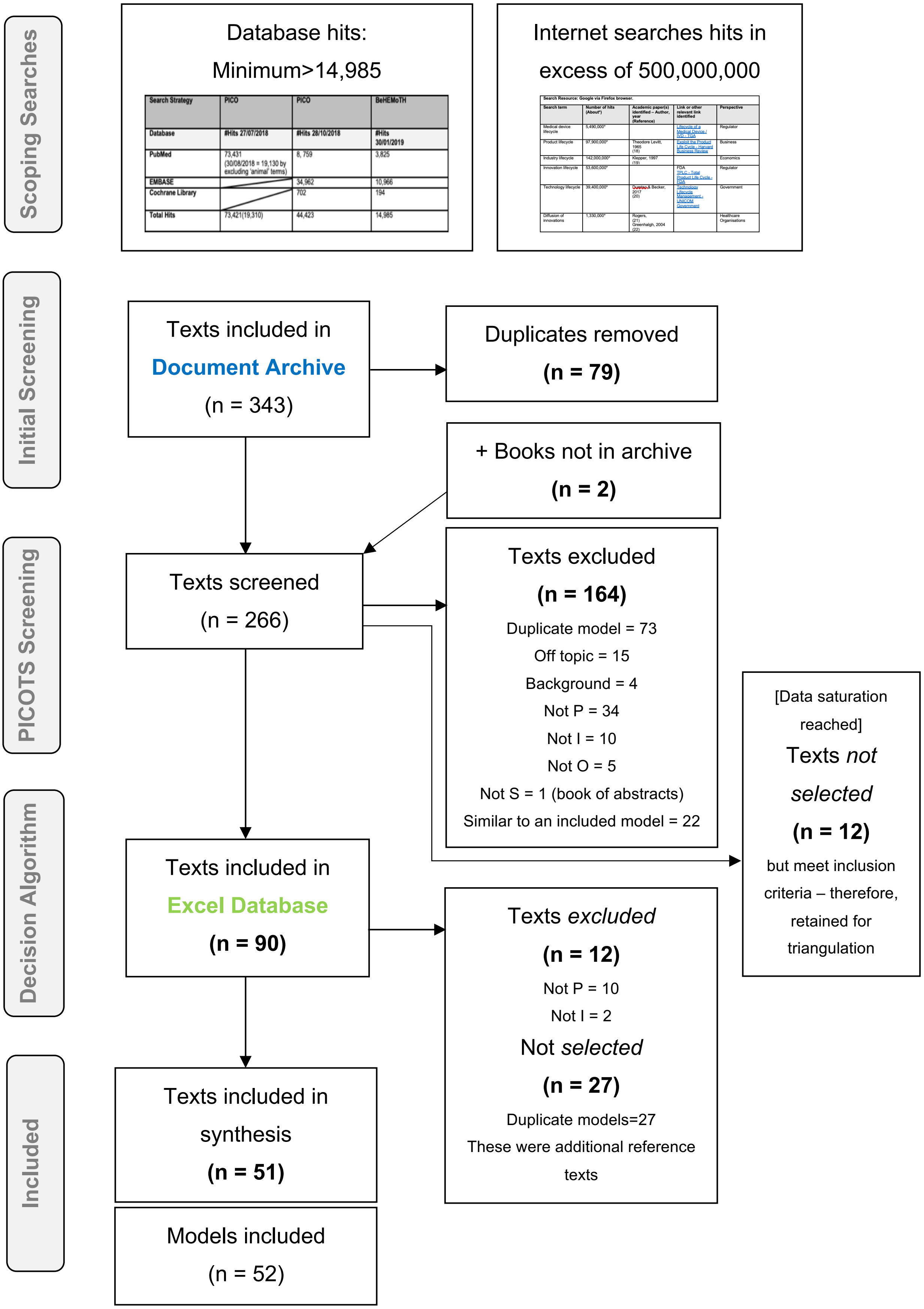

Supplement: Harkin et al. supplementary material 4 — Harkin et al. supplementary material [file S026646232300274Xsup004.pdf]
