# Supplementary material for: Lifecycle evaluation of medical devices: supporting or jeopardizing patient outcomes? A comparative analysis of evaluation models
Source: Int J Technol Assess Health Care. 2024 Jan 5;40(1):e2. doi: 10.1017/S026646232300274X (PMC10859834; doi:10.1017/S026646232300274X)
Supplement: Harkin et al. supplementary material 5 — Harkin et al. supplementary material [file S026646232300274Xsup005.pdf]

LIFECYCLE EVALUATION OF MEDICAL DEVICES – SUPPORTING OR JEOPARDIZING PATIENT OUTCOMES? A COMPARATIVE ANALYSIS OF EVALUATION MODELS

Authors: Kathleen Harkin, ORCID ID https://orcid.org/0000-0003-3260-9059; Jan Sorensen, ORCID ID https://orcid.org/0000-0003-0857-9267; Steve Thomas, ORCID ID https://orcid.org/0000-0001-9306-0114

Harkin_Supplemental-5_List of models & Categorization

List of Models

Table 1: Models included in the synthesis.

Model name given by Author	Also known as	*(Abbreviated) Model Name*	Author Year (Ref)
How to introduce a new product	-	*Baldock-NPD*	Baldock 1960 (1)
Life of an innovation	Diffusion of Innovations	*DOI*	Rogers 1963 (2)
A New Product Growth Model for Consumer Durables	Bass Model	*Bass*	Bass 1963, published 1969 (3)
Product Life Cycle	-	*PLC*	Levitt 1965 (4), but originally postulated by Kuznets in 1930 (5).
7 Stages in the Career of a Medical Innovation	-	*7Sm-IC*	McKinlay 1981 (6)
How innovations become routinized	-	*IRP*	Yin 1981 (7)
Stages of the New Product Development Process	BAH Model	*BAH-NPD*	Booz, Hamilton & Allen 1982 (8) (unavailable so we drew on Fortenberry’s summary (9))
The stages of growth: Organization development model	Business Life Cycle	*BLC*	Galbraith 1982 (10)
The five stages of new product industries	Industry Life Cycle	*ILC*	Gort & Klepper 1982 (11)
A *skeleton* of the new product process	New Product Development Process or New product process activities	*CK-NPD*	Cooper & Kleinschmidt 1986 (12)
A Diffusion Theory of Adoption and Substitution for Successive Generations of High-Technology Products	Norton-Bass Model	*Norton-Bass*	Norton & Bass 1987 (13)

A stage-gate system for managing new products	Stage-gate system	*SG-CK-NPD*	Cooper 1990 (14)
Technology Adoption Life Cycle	The High-Tech Marketing Model	*TALC*	Moore 1991 & 2005 (15)
Generalized Bass Model	GBM	*G-Bass-M*	Bass, Krishnan, & Jain 1994 (16)
Technology Readiness Levels	-	*TRL*	Mankins 1995 (17)
Design controls guidance for medical device manufacturers	-	*MDDP*	FDA 1997 (18)
Technology Transfer 'GATEWAY' chart	Veteran Affairs Research Development & Technology Transfer Process	*VA-NPD*	Sheredos & Cupo 1997 (19)
Four stages of economic evaluation	Iterative economic evaluation (across the health technology lifecycle)	*4S-IEE*	Sculpher, Drummond & Buxton 1997 (20)
Reach, Efficacy – Adoption, Implementation, Maintenance (RE-AIM)	RE-AIM	*RE-AIM*	Glasgow, Vogt & Boles 1999 (21)
Major phases in the life span of a medical device	-	*MDLS*	Cheng 2003 (22)
Healthcare Technology Life Cycle	-	*HCTLC*	Cheng 2003 (22)
A Systems-based User-centred Approach to Healthcare Design	-	*SUHCD*	Clarkson et al., 2004 (23)
Conceptual Model for Considering the Determinants of Diffusion, Dissemination, and Implementation of Innovations in Health Service Delivery and Organizations	-	*DDDII*	Greenhalgh et al., 2004 (24,25)
Total Product Life Cycle (Composed of the Marketing Development Life Cycle, the Device Scientific Life Cycle, and the Device Regulatory Life Cycle)	TPLC	*TPLC*	Feigal 2000 (FDA) (26)

Technology Adoption Life Cycle Conceptual Attractor H Framework	H-Framework	*TALC-CAHF*	Meade & Rabelo 2004 (27)
Equipment Life Cycle	Healthcare Technology Management Cycle	*ELC*	Worm 2015 (28) (Described by Temple-Bird et al in 2005 as the Healthcare Technology Management Cycle (29))
Technology Risk and Readiness Assessment Framework		*IRM-TRL*	Mankins 2009 (30)
IDEAL Framework	IDEAL	*IDEAL*	McCulloch et al., 2009 (31–33)
Industrial Emergence Framework	Technology Roadmapping	*IEF*	Phaal et al., 2009 (34)
Stage-gate process for the development of medical devices	Medical Device Development: High-level Representation of Development Phases and of Functional Activities	*SG-MDDP*	Pietzsch et al., 2009 (35)
The Innovation Cycle	-	*IC+*	Croslin 2010 (36)
Lifecycle of Technology	-	*TLC*	Mytton et al., 2010 (37)
EAES recommendations on methodology of innovation management in endoscopic surgery	-	*EIM-2DA*	Neugebauer & Becker et al., 2010 (38)
A framework for successful new product development	-	*Bhuiyan-NPD*	Bhuiyan 2011 (39)
Conceptual Framework of the University Spin-off Venturing Process	-	*USVP*	Rasmussen 2011 (40)
The four phases of the medical device life-cycle	Medical device life-cycle	*MDLC*	Velasquez-Berumen 2011 (41)
Health Canada's Lifecycle Approach to Health Product Vigilance & Product Vigilance and Benefit-Risk Management Cycle	-	*HCanada-MDRegLC*	Health Canada 2013 (42)
The Innovation Cycle	-	*IC*	CIRAS 2013 (43)
The Innovation Cycle	-	*WW-IC*	Wright & Weinstein 2013 (44)

The integration of risk management process with the lifecycle of medical device software	-	*IRM-SaMDDP*	Pecoraro & Luzi 2014 (45)
A tool for distinguishing between experimental, innovative and established treatment	Treatment 'lifecycle' framework	*RxLCF*	Provoost et al., 2014 (46)
Product Innovation Life Cycle	-	*PrLC*	EU Procurement Directive approach to procuring innovative products, presented by An Baeyens 2016 (47)
IDEAL-D	-	*IDEAL-D*	Sedrakyan et al., 2016 (48)
Framework for Theorizing and Evaluating Non-adoption, Abandonment, and Challenges to the Scale-up, Spread and Sustainability of Health and Care Technologies	NASSS Framework	*NASSS*	Greenhalgh et al., 2017 (49)
Health Technology Life Cycle	-	*HTLC*	Gutiérrez-Ibarluzea, Chiumente & Dauben 2017 (50)
Optimal methodology for the introduction of new orthopaedic implants	-	*OIM-DA*	Hannan et al., 2017 (51)
NASA Program/Project Life Cycle Process (NASA)	-	*PrLC*	NASA 2017 (52)
New Health Technologies – Lifecycle for integration into healthcare systems	-	*nHTLC4I*	Paris et al., 2017 (53)
Life cycle of medical devices - Lifecycle approach to regulation	-	*TGA-MDRegLC*	Reeves & Garcia 2014 (TGA) 2017 (54)
EUnetHTA's lifecycle approach	-	*EUnetHTA-MDLC*	Meyer, Brühl & Ormstad 2018 (55)
The Device Development Process	Total Product Life Cycle	*FDA-MDRegLC*	FDA (56)
Lifecycle of a medical device	-	*Swissmedic-MDRegLC*	Swissmedic 2018 (57)

Categorizing perspectives

The following table shows how individual study/model perspectives were grouped into four broad categories.

**Trade & Industry (19)**	**Policymaking (16)**
Academia & Marketing	Academia, Research, Economics, Sociology & Agriculture
Business	Academic
Business & Academia	Access, Value & Sustainability
Business & Marketing	EU DG GROW
Business Management	Health systems policymaking
Business Strategy	Healthcare & Health economics
Economics	Healthcare & research
Industry	HTA
Manufacturing Industry	HTA & Economists
Marketing	Public Health
Medical device design engineers	Sociology and agricultural policymaking
Procurement	Space & Aeronautics
Software Engineering	Systems Engineering
**Healthcare (9)**	**Regulation (8)**
Biomedical Engineering & Healthcare Management	Healthcare/ Regulatory
Clinical	Regulatory
Engineering & Safety	
Healthcare Access	
Surgical	

Model categorization

**Trade & Industry (n=19)**	**Policymaking (n=16)**
PLC(4)	*DOI*(2)
Baldock-NPD(1)	*IRP*(7)
Bass(58,59)	*7Sm-IC*(6)
BAH-NPD(8,9)	*TRL*(17)
BLC(10)	*4S-IEE*(20,64)
ILC(11,60,61)	*RE-AIM*(21)
CK-NPD(12)	*DDDII*(24)
Norton-Bass(13)	*IRM-TRL*(30)
SG-CK-NPD(14)	*TLC*(37)
TALC(15,62)	*MDLC*(41)
G-Bass-M(16)	*PrLC*(52)
TALC-CAHF(27)	*PILC*(47)
USVP(40)	*NASSS*(49)
IEF(34,63)	*HTLC*(50)
SG-MDDP(35)	*nHTLC4I*(65)
IC+(36)	*EUnetHTA-MDLC*(55)
Bhuiyan-NPD(39)	
IC(43)	
IRM-SaMDDP(45)	
**Healthcare (n=9)**	**Regulation (n=8)**
VA-NPD(19)	*MDDP*(18)
SUHCD(23)	*TPLC*(26,69–71)
ELC(28,29)	*HCTLC*(22)
IDEAL(31,32,66)	*MDLS*(22)
EIM-2DA(38)	*HCanada-MDRegLC*(42,72)
WW-IC(44)	*TGA-MDRegLC*(54)
RxLCF(46)	*Swissmedic-MDRegLC*(57)
IDEAL-D(48,67,68)	*FDA-MDRegLC*(56)
OIM-DA(51)	

Model categorization with Author name

Trade & Industry (n=19)	Policymaking (n=16)
PLC, *Levitt, 1965*(4)	***DOI,*** *Rogers, 1962*(2)
Baldock-NPD, *Baldock, 1960*(1)	***IRP,*** *Yin, 1981*(7)
Bass, *Bass, 1969*(58,59)	***7SMI career,*** *McKinlay, 1981*(6)
BAH-NPD, *Booz, Allen & Hamilton, 1982*(8,9)	***TRL,*** *Mankins, 1995*(17)
BLC, *Galbraith, 1982*(10)	***4S-IEE,*** *Sculpher, Buxton, & Drummond, 1997*(20,64)
ILC, *Gort & Klepper, 1982*(11,60,61)	***RE-AIM,*** *Glasgow, Vogt, & Boles, 1999*(21)
CK-NPD, *Cooper & Kleinschmidt, 1986*(12)	***DDDII,*** *Greenhalgh et al., 2004*(24)
Norton-Bass, *Norton & Bass, 1987*(13)	***IRM-TRL,*** *Mankins, 2009*(30)
SG-CK-NPD, *Cooper, 1990*(14)	***TLC,*** *Mytton et al., 2010*(37)
TALC, *Moore, 2001*(15,62)	***MDLC,*** *Velazquez-Berumen, 2011*(41)
GBM, *Bass, Krishnan, & Jain, 1994*(16)	***PrLC,*** *NASA, 2014*(52)
TALC-CAHF, *Meade & Rabelo, 2004*(27)	***PILC,*** *Baeyens, 2016*(47)
USVP, *Rasmussen, 2011*(40)	***NASSS,*** *Greenhalgh et al., 2017*(49)
IEF, *Phaal et al., 2009*(34,63)	***HTLC,*** *Gutiérrez-Ibarluzea, Chiumente & Dauben, 2017*(50)
SG-MDDP, *Pietzsch et al., 2009*(35)	***nHTLC4I,*** *Paris et al., 2017*(65)
IC+, *Croslin, 2010*(36)	***EUnetHTA-MDLC,*** *Meyer, Brühl & Omstad, 2018*(55)
Bhuiyan-NPD, *Bhuiyan, 2011*(39)	
IC, *CIRAS, 2013*(43)	
IRM-SaMDDP, *Pecoraro & Luzi, 2017*(45)	
**Healthcare (n=9)**	**Regulation (n=8)**
VA-NPD, *Sheredos & Cupo, 1997*(19)	***MDDP,*** *FDA, 1997(18)*
SUHCD, *Clarkson et al., 2004*(23)	***TPLC,*** *Feigal Jr. for the Institute of Medicine, 2010*(26,69–71)
ELC, *Worm (THET), 2015*(28,29)	***HCTLC,*** *Cheng, 2003*(22)
IDEAL, *McCulloch et al., 2009*(31,32,66)	***MDLS,*** *Cheng, 2003*(22)
EIM-2DA, *Neugebauer & Becker et al., 2010*(38)	***HCanada-MDRegLC,*** *Health Canada, 2013*(42,72)
WW-IC, *Wright & Weinstein, 2013*(44)	***TGA-MDRegLC,*** *Reeves & Garcia, 2014*(54)
RxLCF, *Provoost et al., 2014*(46)	***FDA-MDRegLC,*** *FDA, 2018*(56)
IDEAL-D, *Pennell et al., 2016*(48,67,68)	***Swissmedic-MDRegLC,*** *Swissmedic, 2017*(57)
OIM-DA, *Hannan et al., 2017*(51)	

References

Note – these include additional references to those listed in Supplemental-6_List of key reference texts.

1. Baldock AL. How to introduce a new product. In: 1960 EI National Conference on the Application of Electrical Insulation. 1960. p. 171–2.

2. Rogers EM. Diffusion of innovations. 3rd ed. New York : London: Free Press ; Collier Macmillan; 1983. 453 p.

3. Bass FM. A new product growth for model consumer durables. Manage Sci. 1969;15(5):215–27.

4. Levitt T. Exploit the Product Life Cycle. Harv Bus Rev. 1965 Nov 1;43(6):81–94.

5. Kuznets S. Secular movements in production and prices: Their nature and their bearing upon cyclical fluctuations. A. M. Kelley; 1967. 576 p.

6. McKinlay JB. From promising report to standard procedure: Seven stages in the career of a medical innovation. Milbank Mem Fund Q Health Soc. 1981;59(3):374–411.

7. Yin RK. Life histories of innovations: How new practices become routinized. Public Adm Rev. 1981;41(1):21–8.

8. Booz, Allen, Hamilton. New products management for the 1980s. New York: Booz, Allen and Hamilton Inc; 1982. 24 p.

9. Fortenberry Jr JL. Booz, Allen, and Hamilton's new product process. In: John L Fortenberry Jr Nonprofit Marketing [Internet]. Jones & Bartlett Publishers; 2013. p. 11–8. Available from: https://books.google.ie/books?id=0AFwbnQrm7wC&printsec=frontcover&source=gbs_ge_summary_r&cad=0#v=onepage&q&f=false

10. Galbraith J. The stages of growth. J Bus Strategy. 1982 Summer;3(000001):70–9.

11. Gort M, Klepper S. Time paths in the diffusion of product innovations. Econ J (London). 1982 Sep;92(367):630.

12. Cooper RG, Kleinschmidt EJ. An investigation into the new product process: Steps, deficiencies, and impact. J Prod Innov Manag. 1986 Jun 1;3(2):71–85.

13. Norton JA, Bass FM. A diffusion theory model of adoption and substitution for successive generations of high-technology products. Manage Sci. 1987 Sep 1;33(9):1069–87.

14. Cooper RG. Stage-gate systems: A new tool for managing new products. Bus Horiz. 1990 May 1;33(3):44–54.

15. Moore GA. Crossing the chasm: Marketing and selling high-tech products to mainstream customers. New York: PerfectBound; 2001.

16. Bass FM, Krishnan TV, Jain DC. Why the Bass Model fits without decision variables. Mark Sci. 1994 Aug;13(3):203–23.

17. Mankins JC. Technology Readiness Levels. A White Paper [Internet]. Advanced Concepts Office, Office of Space Access and Technology, NASA; 1995. Available from: https://aiaa.kavi.com/apps/group_public/download.php/2212/TRLs_Mankins%20Paper_1995.pdf

18. Center for Devices and Radiological Health. Design control guidance for medical device manufacturers [Internet]. USA: Food and Drug Administration (FDA); 1997 Mar [cited 2020 May 14] p. 53. Available from: https://www.fda.gov/media/116573/download

19. Sheredos SJ, Cupo ME. The Department of Veterans Affairs Rehabilitation Research and Development Service's Technology Transfer Process. Technol Disabil. 1997;7(1–2):25–9.

20. Sculpher M, Drummond M, Buxton M. The iterative use of economic evaluation as part of the process of health technology assessment. J Health Serv Res Policy. 1997 Jan;2(1):26–30.

21. Glasgow RE, Vogt T, Boles S. Evaluating the public health impact of health promotion interventions: The RE-AIM framework. Am J Public Health. 1999 Sep;89(9):1322–7.

22. Cheng M. Medical device regulations: Global overview and guiding principles. Geneva: World Health Organization; 2003. 43 p.

23. Clarkson PJ, Buckle P, Coleman R, Stubbs D, Ward J, Jarrett J, et al., Design for patient safety: A review of the effectiveness of design in the UK health service. J Eng Des. 2004 Apr;15(2):123–40.

24. Greenhalgh T, Robert G, Macfarlane F, Bate P, Kyriakidou O. Diffusion of innovations in service organizations: Systematic review and recommendations. Milbank Q. 2004;82(4):581–629.

25. Greenhalgh T, Robert G, Bate P, Kyriakidou O, MacFarlane F, Peacock R. How to Spread Good Ideas: A systematic review of the literature on diffusion, dissemination and sustainability of innovations in health service delivery and organisation. London, England; 2004 Apr p. 427. (Report for the National Co-ordinating Centre for NHS Service Delivery and Organisation R&D (NCCSDO)).

26. Feigal D. Total Product Life Cycle [Internet]. Online presented at; 2003 Jan 16 [cited 2021 Jun 1]. Available from: https://webarchive.library.unt.edu/eot2008/20090120125024/http://www.fda.gov/cdrh/strategic/presentations/tplc.ppt

27. Meade PT, Rabelo L. The technology adoption life cycle attractor: Understanding the dynamics of high-tech markets. Technol Forecast Soc Change. 2004 Sep;71(7):667–84.

28. Worm A. Managing the lifecycle of medical equipment [Internet]. London, England: Tropical Health and Education Trust (THET); 2015 Sep [cited 2018 Jan 9] p. 12. Available from: https://www.thet.org/wp-content/uploads/2017/08/THET_Managing_the_medical_equipment_lifecycle_LOW-RES.pdf

29. Temple-Bird C, Kawohl W, Lenel A, Kaur M. How to plan and budget for your healthcare technology. St. Albans: Teaching-aids At Low Cost; 2006. 299 p. ('How to Manage' Series for Healthcare Technology).

30. Mankins JC. Technology readiness and risk assessments: A new approach. Acta Astronaut. 2009 Nov;65(9–10):1208–15.

31. Ergina PL, Cook JA, Blazeby JM, Boutron I, Clavien PA, Reeves BC, et al., Challenges in evaluating surgical innovation. Lancet. 2009 Sep 26;374(9695):1097–104.

32. Barkun JS, Aronson JK, Feldman LS, Maddern GJ, Strasberg SM. Evaluation and stages of surgical innovations. Lancet. 2009 Sep 26;374(9695):1089–96.

33. McCulloch P, Altman DG, Campbell WB, Flum DR, Glasziou P, Marshall JC, et al., No surgical innovation without evaluation: The IDEAL recommendations. Lancet. 2009 Sep 26;374(9695):1105–12.

34. Phaal R, O'Sullivan E, Farrukh C, Probert D. Developing a framework for mapping industrial emergence. In: PICMET '09 - 2009 Portland International Conference on Management of Engineering Technology. 2009. p. 428–40.

35. Pietzsch JB, Shluzas LA, Paté-Cornell ME, Yock PG, Linehan JH. Stage-gate process for the development of medical devices. J Med Devices. 2009 Jun 1;3(2):021004.

36. Croslin D. Innovate the future: A radical new approach to IT innovation. Prentice Hall; 2010. 281 p.

37. Mytton OT, Velazquez A, Banken R, Mathew JL, Ikonen TS, Taylor K, et al., Introducing new technology safely. Qual Saf Health Care. 2010 Aug;19 Suppl 2:i9-14.

38. Neugebauer EAM, Becker M, Buess GF, Cuschieri A, Dauben HP, Fingerhut A, et al., EAES recommendations on methodology of innovation management in endoscopic surgery. Surg Endosc. 2010 Jul;24(7):1594–615.

39. Bhuiyan N. A framework for successful new product development. J Ind Eng Manag. 2011 Dec 11;4(4):746–70.

40. Rasmussen E. Understanding academic entrepreneurship: Exploring the emergence of university spin-off ventures using process theories. Int Small Bus J. 2011 Oct 1;29(5):448–71.

41. World Health Organization. Development of medical device policies [Internet]. Geneva, Switzerland: World Health Organization; 2011. 39 p. (WHO medical device technical series). Available from: http://apps.who.int/medicinedocs/documents/s21559en/s21559en.pdf?ua=1

42. Health Canada. Health product vigilance framework. [Internet]. Ottawa, Ontario: Health Canada; 2013 [cited 2019 Oct 14]. Available from: http://ra.ocls.ca/ra/login.aspx?inst=centennial&url=https://www.deslibris.ca/ID/235384

43. Extension and Outreach, College of Engineering. CIRAS innovation cycle brochure [Internet]. Iowa State University; 2013. Available from: www.ciras.iastate.edu

44. Wright JG, Weinstein S. The Innovation Cycle: A Framework for taking surgical innovation into clinical practice. J Bone Jt Surg. 2013 Nov 6;95(21):e164.

45. Pecoraro F, Luzi D. The integration of the risk management process with the lifecycle of medical device software. Methods Inf Med. 2014;53(2):92–8.

46. Provoost V, Tilleman K, D'Angelo A, De Sutter P, de Wert G, Nelen W, et al., Beyond the dichotomy: A tool for distinguishing between experimental, innovative and established treatment. Hum Reprod. 2014 Mar;29(3):413–7.

47. Baeyens A. How the new Procurement Directive may contribute to spur innovative purchases in the health sector. Launch of the IMDA Strategy 2016-2020 Dublin; 2016 Feb 11; Royal College of Physicians, 6 Kildare Street, Dublin 2.

48. Sedrakyan A, Campbell B, Merino JG, Kuntz R, Hirst A, McCulloch P. IDEAL-D: A rational framework for evaluating and regulating the use of medical devices. BMJ. 2016 Jun 9;353:i2372.

49. Greenhalgh T, Wherton J, Papoutsi C, Lynch J, Hughes G, A'Court C, et al., Beyond adoption: A new framework for theorizing and evaluating nonadoption, abandonment, and challenges to the scale-up, spread, and sustainability of health and care technologies. J Med Internet Res. 2017 01;19(11):e367.

50. Gutiérrez-Ibarluzea I, Chiumente M, Dauben HP. The life cycle of health technologies. Challenges and ways forward. Front Pharmacol. 2017 Jan 24;8(Article 14):1–4.

51. Hannan R, Arora V, Beaver R, Harvie P. How should new orthopaedic implants be introduced: An example and recommendations for best practice. ANZ J Surg. 2017 Nov 9;88(4):284–9.

52. NASA Office of the Chief Engineer (OCE). NASA Systems Engineering Handbook [Internet]. Rev 2. Washington, D.C: National Aeronautics and Space Administration (NASA); 2016 [cited 2018 Nov 26]. 356 p. Available from: https://ntrs.nasa.gov/archive/nasa/casi.ntrs.nasa.gov/20170001761.pdf

53. Paris V, Slawomirski L, Colbert A. Ensuring timely and affordable access to medical devices. In: OECD. New Health Technologies: Managing Access, Value and Sustainability [Internet]. OECD Publishing; 2017 [cited 2020 Jan 24]. p. 117–58. Available from: https://doi.org/10.1787/9789264266438-7-en

54. Reeves O, Garcia J. Life cycle of medical devices: Lifecycle approach to regulation & the importance of reporting incidents to the TGA [Internet]. Conference presentation presented at: Australian Biomedical Engineering Conference; 2014 Aug 20; National Convention Centre Canberra. Available from: https://www.tga.gov.au/presentation-life-cycle-medical-devices

55. JA3 Work Package 5 – Lifecycle Approach to improve Evidence Generation – EUnetHTA [Internet]. [cited 2021 May 9]. Available from: https://eunethta.eu/ja3-archive/work-package-5-life-cycle-approach-to-improve-evidence-generation/

56. Office of the Commissioner. FDA. FDA; 2020 [cited 2021 Apr 28]. The Device Development Process. Available from: https://www.fda.gov/patients/learn-about-drug-and-device-approvals/device-development-process

57. Swissmedic 2017 © Copyright. Guide to the regulation of medical devices [Internet]. 2017 [cited 2018 Jul 16]. Available from: https://www.swissmedic.ch/swissmedic/en/home/medical-devices/regulation-of-medical-devices/medical-device-regulation_online-guide.html

58. Bass FM. A new product growth for model consumer durables. Manage Sci. 2004 Dec;50(12_supplement):1825–32.

59. Bass FM. A dynamic model of market share and sales behavior. In: Winter Conference [Internet]. Chicago, IL; 1963 [cited 2020 Jan 19]. p. 263–76. Available from: http://www.bassbasement.org/F/N/FMB/Pubs/Bass%201963%20Dynamic%20Model.pdf

60. Klepper S. Industry life cycles. Ind Corp Chang. 1997 Jan 1;6(1):145–82.

61. Klepper S. Entry, exit, growth, and innovation over the Product Life Cycle. Am Econ Rev. 1996;86(3):562–83.

62. Balakrishnan S. Strategies for High-Tech Markets. Based on Inside the Tornado (by Geoffrey A. Moore) [Internet]. Lecture presented at; [cited 2020 Jan 14]; University of

Washington. Available from: http://faculty.washington.edu/sundar/NPM/LECTURE-PDF/S%2316b-TALC-Handout.pdf

63. Phaal R, O'Sullivan E, Routley M, Ford S, Probert D. A framework for mapping industrial emergence. Technol Forecast Soc Change. 2011 Feb;78(2):217–30.

64. Sculpher MJ, Claxton K, Drummond M, McCabe C. Whither trial-based economic evaluation for health care decision making? Health Econ. 2006 Jul;15(7):677–87.

65. OECD. New health technologies: Managing access, value and sustainability [Internet]. Paris: OECD Publishing; 2017 [cited 2018 Aug 8]. 228 p. Available from: http://www.oecd-ilibrary.org/social-issues-migration-health/new-health-technologies/new-health-technologies-managing-access-value-and-sustainability_9789264266438-4-en

66. McCulloch P, Hirst, Maruthappu M. A plan for whole-life-cycle scientific evaluation of surgical innovation: The Idea, Development, Exploration, Assessment, Long-term study (IDEAL) recommendations. J Am Coll Surg. 2014;219(4):e160.

67. Pennell CP, Hirst AD, Campbell WB, Sood A, Agha RA, Barkun JST, et al., Practical guide to the Idea, Development and Exploration stages of the IDEAL Framework and Recommendations. Br J Surg. 2016 Apr;103(5):607–15.

68. Pennell CP, Hirst A, Sedrakyan A, McCulloch PG. Adapting the IDEAL Framework and Recommendations for medical device evaluation: A modified Delphi survey. Int J Surg. 2016 Apr;28:141–8.

69. Strategic Plan - Total Product Life Cycle Model [Internet]. [cited 2021 May 25]. Available from: https://webarchive.library.unt.edu/eot2008/20081023160331/http://www.fda.gov/cdrh/strategic/tplc.html

70. Hausman ED, Altaie SS. Regulatory aspects of total product life cycle. Diabetes Technol Ther. 2004 Dec;6(6):761–6.

71. Feigal DW. Appendix D Impact of the regulatory framework on medical device development and innovation. In: Wizemann T, editor. Public Health Effectiveness of the FDA 510(k) Clearance Process: Balancing Patient Safety and Innovation: Workshop Report [Internet]. Washington (DC): National Academies Press (US); 2010 [cited 2020 Jan 20]. p. 113–28. Available from: http://www.ncbi.nlm.nih.gov/books/NBK209791/

72. Therapeutic Goods Administration. Therapeutic product vigilance [Internet]. Australia: Therapeutic Goods Administration, Department of Health; 2017 p. 15. Report No.: v1.4 September 2017. Available from: https://www.tga.gov.au/publication/therapeutic-product-vigilance
