# Supplementary material for: Lifecycle evaluation of medical devices: supporting or jeopardizing patient outcomes? A comparative analysis of evaluation models
Source: Int J Technol Assess Health Care. 2024 Jan 5;40(1):e2. doi: 10.1017/S026646232300274X (PMC10859834; doi:10.1017/S026646232300274X)
Supplement: Harkin et al. supplementary material 6 — Harkin et al. supplementary material [file S026646232300274Xsup006.pdf]

LIFECYCLE EVALUATION OF MEDICAL DEVICES – SUPPORTING OR JEOPARDIZING PATIENT OUTCOMES? A COMPARATIVE ANALYSIS OF EVALUATION MODELS

Authors: Kathleen Harkin, ORCID ID https://orcid.org/0000-0003-3260-9059; Jan Sorensen, ORCID ID https://orcid.org/0000-0003-0857-9267; Steve Thomas, ORCID ID https://orcid.org/0000-0001-9306-0114

Harkin_Supplemental-6_List of key reference texts

List of key[1] references of models included in the synthesis

SR-1. Baldock AL. How to introduce a new product. In: 1960 EI National Conference on the Application of Electrical Insulation. 1960. p. 171–2.

SR-2. Rogers EM. Diffusion of innovations. 3rd ed. New York : London: Free Press ; Collier Macmillan; 1983. 453 p.

SR-3. Bass FM. A new product growth for model consumer durables. Manage Sci. 1969;15(5):215–27.

SR-4. Levitt T. Exploit the Product Life Cycle. Harv Bus Rev. 1965 Nov 1;43(6):81–94.

SR-5. McKinlay JB. From promising report to standard procedure: Seven stages in the career of a medical innovation. Milbank Mem Fund Q Health Soc. 1981;59(3):374–411.

SR-6. Yin RK. Life histories of innovations: How new practices become routinized. Public Adm Rev. 1981;41(1):21–8.

SR-7. Fortenberry Jr JL. Booz, Allen, and Hamilton's new product process. In: John L Fortenberry Jr Nonprofit Marketing [Internet]. Jones & Bartlett Publishers; 2013. p. 11–8. Available from: https://books.google.ie/books?id=0AFwbnQrm7wC&printsec=frontcover&source=gbs_ge_summary_r&cad=0#v=onepage&q&f=false

SR-8. Galbraith J. The stages of growth. J Bus Strategy. 1982 Summer;3(000001):70–9.

SR-9. Gort M, Klepper S. Time paths in the diffusion of product innovations. Econ J (London). 1982 Sep;92(367):630.

SR-10. Cooper RG, Kleinschmidt EJ. An investigation into the new product process: Steps, deficiencies, and impact. J Prod Innov Manag. 1986 Jun 1;3(2):71–85.

SR-11. Norton JA, Bass FM. A diffusion theory model of adoption and substitution for successive generations of high-technology products. Manage Sci. 1987 Sep 1;33(9):1069–87.

SR-12. Cooper RG. Stage-gate systems: A new tool for managing new products. Bus Horiz. 1990 May 1;33(3):44–54.

SR-13. Moore GA. Crossing the chasm: Marketing and selling high-tech products to mainstream customers. New York: PerfectBound; 2001.

SR-14. Bass FM, Krishnan TV, Jain DC. Why the Bass Model fits without decision variables. Mark Sci. 1994 Aug;13(3):203–23.

[1] These are the references chosen to represent each model as the key reference text, but they're not the only references for the models. Some additional references are listed in Supplemental-5_List of models & Categorization.

SR-15. Mankins JC. Technology Readiness Levels. A White Paper [Internet]. Advanced Concepts Office, Office of Space Access and Technology, NASA; 1995. Available from: https://aiaa.kavi.com/apps/group_public/download.php/2212/TRLs_Mankins%20Paper_1995.pdf

SR-16. Center for Devices and Radiological Health. Design control guidance for medical device manufacturers [Internet]. USA: Food and Drug Administration (FDA); 1997 Mar [cited 2020 May 14] p. 53. Available from: https://www.fda.gov/media/116573/download

SR-17. Sheredos SJ, Cupo ME. The Department of Veterans Affairs Rehabilitation Research and Development Service's Technology Transfer Process. Technol Disabil. 1997;7(1–2):25–9.

SR-18. Sculpher M, Drummond M, Buxton M. The iterative use of economic evaluation as part of the process of health technology assessment. J Health Serv Res Policy. 1997 Jan;2(1):26–30.

SR-19. Glasgow RE, Vogt T, Boles S. Evaluating the public health impact of health promotion interventions: The RE-AIM framework. Am J Public Health. 1999 Sep;89(9):1322–7.

SR-20. Cheng M. Medical device regulations: Global overview and guiding principles. Geneva: World Health Organization; 2003. 43 p.

SR-21. Clarkson PJ, Buckle P, Coleman R, Stubbs D, Ward J, Jarrett J, et al. Design for patient safety: A review of the effectiveness of design in the UK health service. J Eng Des. 2004 Apr;15(2):123–40.

SR-22. Greenhalgh T, Robert G, Macfarlane F, Bate P, Kyriakidou O. Diffusion of innovations in service organizations: Systematic review and recommendations. Milbank Q. 2004;82(4):581–629.

SR-23. Feigal D. Total Product Life Cycle [Internet]. Online presented at; 2003 Jan 16 [cited 2021 Jun 1]. Available from: https://webarchive.library.unt.edu/eot2008/20090120125024/http://www.fda.gov/cdrh/strategic/presentations/tplc.ppt

SR-24. Meade PT, Rabelo L. The technology adoption life cycle attractor: Understanding the dynamics of high-tech markets. Technol Forecast Soc Change. 2004 Sep;71(7):667–84.

SR-25. Worm A. Managing the lifecycle of medical equipment [Internet]. London, England: Tropical Health and Education Trust (THET); 2015 Sep [cited 2018 Jan 9] p. 12. Available from: https://www.thet.org/wp-content/uploads/2017/08/THET_Managing_the_medical_equipment_lifecycle_LOW-RES.pdf

SR-26. Mankins JC. Technology readiness and risk assessments: A new approach. Acta Astronaut. 2009 Nov;65(9–10):1208–15.

SR-27. McCulloch P, Altman DG, Campbell WB, Flum DR, Glasziou P, Marshall JC, et al. No surgical innovation without evaluation: The IDEAL recommendations. Lancet. 2009 Sep 26;374(9695):1105–12.

SR-28. Phaal R, O'Sullivan E, Farrukh C, Probert D. Developing a framework for mapping industrial emergence. In: PICMET '09 - 2009 Portland International Conference on Management of Engineering Technology. 2009. p. 428–40.

SR-29. Pietzsch JB, Shluzas LA, Paté-Cornell ME, Yock PG, Linehan JH. Stage-gate process for the development of medical devices. J Med Devices. 2009 Jun 1;3(2):021004.

SR-30. Croslin D. Innovate the future: A radical new approach to IT innovation. Prentice Hall; 2010. 281 p.

SR-31. Mytton OT, Velazquez A, Banken R, Mathew JL, Ikonen TS, Taylor K, et al. Introducing new technology safely. Qual Saf Health Care. 2010 Aug;19 Suppl 2:i9-14.

SR-32. Neugebauer EAM, Becker M, Buess GF, Cuschieri A, Dauben HP, Fingerhut A, et al. EAES recommendations on methodology of innovation management in endoscopic surgery. Surg Endosc. 2010 Jul;24(7):1594–615.

SR-33. Bhuiyan N. A framework for successful new product development. J Ind Eng Manag. 2011 Dec 11;4(4):746–70.

SR-34. Rasmussen E. Understanding academic entrepreneurship: Exploring the emergence of university spin-off ventures using process theories. Int Small Bus J. 2011 Oct 1;29(5):448–71.

SR-35. World Health Organization. Development of medical device policies [Internet]. Geneva, Switzerland: World Health Organization; 2011. 39 p. (WHO medical device technical series). Available from: http://apps.who.int/medicinedocs/documents/s21559en/s21559en.pdf?ua=1

SR-36. Health Canada. Health product vigilance framework. [Internet]. Ottawa, Ontario: Health Canada; 2013 [cited 2019 Oct 14]. Available from: http://ra.ocls.ca/ra/login.aspx?inst=centennial&url=https://www.deslibris.ca/ID/235384

SR-37. Extension and Outreach, College of Engineering. CIRAS innovation cycle brochure [Internet]. Iowa State University; 2013. Available from: www.ciras.iastate.edu

SR-38. Wright JG, Weinstein S. The Innovation Cycle: A Framework for taking surgical innovation into clinical practice. J Bone Jt Surg. 2013 Nov 6;95(21):e164.

SR-39. Pecoraro F, Luzi D. The integration of the risk management process with the lifecycle of medical device software. Methods Inf Med. 2014;53(2):92–8.

SR-40. Provoost V, Tilleman K, D'Angelo A, De Sutter P, de Wert G, Nelen W, et al. Beyond the dichotomy: A tool for distinguishing between experimental, innovative and established treatment. Hum Reprod. 2014 Mar;29(3):413–7.

SR-41. Baeyens A. How the new Procurement Directive may contribute to spur innovative purchases in the health sector. Launch of the IMDA Strategy 2016-2020 Dublin; 2016 Feb 11; Royal College of Physicians, 6 Kildare Street, Dublin 2.

SR-42. Pennell CP, Hirst A, Sedrakyan A, McCulloch PG. Adapting the IDEAL Framework and Recommendations for medical device evaluation: A modified Delphi survey. Int J Surg. 2016 Apr;28:141–8.

SR-43. Greenhalgh T, Wherton J, Papoutsi C, Lynch J, Hughes G, A'Court C, et al. Beyond adoption: A new framework for theorizing and evaluating nonadoption, abandonment, and challenges to the scale-up, spread, and sustainability of health and care technologies. J Med Internet Res. 2017 01;19(11):e367.

SR-44. Gutiérrez-Ibarluzea I, Chiumente M, Dauben HP. The life cycle of health technologies. Challenges and ways forward. Front Pharmacol. 2017 Jan 24;8(Article 14):1–4.
SR-45. Hannan R, Arora V, Beaver R, Harvie P. How should new orthopaedic implants be introduced: An example and recommendations for best practice. ANZ J Surg. 2017 Nov 9;88(4):284–9.
SR-46. NASA Office of the Chief Engineer (OCE). NASA Systems Engineering Handbook [Internet]. Rev 2. Washington, D.C: National Aeronautics and Space Administration (NASA); 2016 [cited 2018 Nov 26]. 356 p. Available from: https://ntrs.nasa.gov/archive/nasa/casi.ntrs.nasa.gov/20170001761.pdf
SR-47. Paris V, Slawomirski L, Colbert A. Ensuring timely and affordable access to medical devices. In: OECD. New Health Technologies: Managing Access, Value and Sustainability [Internet]. OECD Publishing; 2017 [cited 2020 Jan 24]. p. 117–58. Available from: https://doi.org/10.1787/9789264266438-7-en
SR-48. Reeves O, Garcia J. Life cycle of medical devices: Lifecycle approach to regulation & the importance of reporting incidents to the TGA [Internet]. Conference presentation presented at: Australian Biomedical Engineering Conference; 2014 Aug 20; National Convention Centre Canberra. Available from: https://www.tga.gov.au/presentation-life-cycle-medical-devices
SR-49. JA3 Work Package 5 – Lifecycle Approach to improve Evidence Generation – EUnetHTA [Internet]. [cited 2021 May 9]. Available from: https://eunethta.eu/ja3-archive/work-package-5-life-cycle-approach-to-improve-evidence-generation/
SR-50. Office of the Commissioner. FDA. FDA; 2020 [cited 2021 Apr 28]. The Device Development Process. Available from: https://www.fda.gov/patients/learn-about-drug-and-device-approvals/device-development-process
SR-51. Swissmedic 2017 © Copyright. Guide to the regulation of medical devices [Internet]. 2017 [cited 2018 Jul 16]. Available from: https://www.swissmedic.ch/swissmedic/en/home/medical-devices/regulation-of-medical-devices/medical-device-regulation_online-guide.html
