# Supplementary material for: Lifecycle evaluation of medical devices: supporting or jeopardizing patient outcomes? A comparative analysis of evaluation models
Source: Int J Technol Assess Health Care. 2024 Jan 5;40(1):e2. doi: 10.1017/S026646232300274X (PMC10859834; doi:10.1017/S026646232300274X)
Supplement: Harkin et al. supplementary material 7 — Harkin et al. supplementary material [file S026646232300274Xsup007.pdf]

LIFECYCLE EVALUATION OF MEDICAL DEVICES – SUPPORTING OR JEOPARDIZING PATIENT OUTCOMES? A COMPARATIVE ANALYSIS OF EVALUATION MODELS

Authors: Kathleen Harkin, ORCID ID https://orcid.org/0000-0003-3260-9059; Jan Sorensen, ORCID ID https://orcid.org/0000-0003-0857-9267; Steve Thomas, ORCID ID https://orcid.org/0000-0001-9306-0114

Harkin_Supplemental-7_Quality appraisal

The Quality Appraisal results are shown in the Table with a tick mark (√) indicating that the model met the criterion, an X indicating that it didn't meet the criterion, or a question mark (?) indicating that it was unclear whether it met the criterion or not. Whether the model by Booz, Allen, and Hamilton met the criteria could not be ascertained (i.e. was unknown) as it was not possible to obtain their book.

Note: The model described by McKinlay[1] is a lifecycle model that he has observed, but not one that he recommends. This is in contrast to all the other models, which are described so that they can be used (not dismissed) to guide strategic planning or specific activities.
He proposes an alternative to the lifecycle model that he has observed – the assessment of population health needs and a thorough assessment of innovations regarding their safety, effectiveness, cost-efficiency, appropriateness, and equity of access prior to allowing them to be publicly funded. He effectively dismisses the lifecycle approach and instead calls for a comprehensive assessment of an innovation before allowed it to be adopted widely.

[1] Through it, he highlights how little evidence is available for medical innovations when they are introduced, adopted by physicians, and later accepted as normal practice by the public and healthcare payers. He argues that this is bad for patients, payers, and healthcare. His model is intended to make us aware of the unacceptable level of evidence available for medical innovations.

Quality appraisal

Author/Proposer, Year of publication	Empirical Data	Explicit literature review	Explicit Theory	*Model*
Baldock, 1960	X	X	X	*Baldock-NPD*
Rogers, 1962	√	?	√	*DOI*
Levitt, 1965	X	X	√	*PLC*
Bass, 1969	√	X	√	*Bass*
McKinlay, 1981	√	?	X	*7Sm-IC*
Yin, 1981	√	X	?	*IRP*
Booz, Allen & Hamilton, 1982	Unknown	Unknown	Unknown	*BAH-NPD*
Galbraith, 1982	X	X	√	*BLC*
Gort & Klepper, 1982	√	X	√	*ILC*
Cooper & Kleinschmidt, 1986	√	X	√	*CK-NPD*
Norton & Bass, 1987	√	X	√	*Norton-Bass*
Cooper, 1990	√	X	√	*SG-CK-NPD*
Bass, Krishnan, & Jain, 1994	√	X	√	*G-Bass-M*
Mankins, 1995	X	X	X	*TRL*
FDA, 1997	X	X	X	*MDDP*
Sculpher, Buxton, & Drummond, 1997	X	X	X	*4S-IEE*
Sheredos & Cupo, 1997	√	X	X	*VA-NPD*
Glasgow, Vogt, & Boles, 1999	X	X	√	*RE-AIM*
Moore, 2001	X	X	√	*TALC*
Cheng, 2003	√	X	?	*MDLS*
Cheng, 2003	X	X	X	*HCTLC*
Clarkson, 2004	√	?	√	*SUHCD*
Greenhalgh et al, 2004	√	√	√	*DDDII*
Meade & Rabelo, 2004	√	X	√	*TALC-CAHF*
Mankins, 2009	X	X	X	*IRM-TRL*
McCulloch et al, 2009	X	X	?	*IDEAL*
Phaal et al, 2009	√	X	√	*IEF*
Pietzsch et al, 2009	√	X	X	*SG-MDDP*
Croslin, 2010	X	X	X	*IC+*
Feigal Jr. for the Institute of Medicine, 2010	X	X	?	*TPLC*
Mytton et al, 2010	X	X	?	*TLC*
Neugebauer & Becker et al, 2010	X	√	√	*EIM-2DA*
Bhuiyan, 2011	X	X	√	*Bhuiyan-NPD*
Rasmussen, 2011	√	X	√	*USVP*
Velazquez-Berumen, 2011	X	X	X	*MDLC*
CIRAS, 2013	X	X	X	*IC*
Health Canada, 2013	X	X	X	*HCanada-MDRegLC*
Wright & Weinstein, 2013	X	X	X	*WW-IC*
Provoost et al, 2014	X	X	X	*RxLCF*
Reeves & Garcia, 2014	X	X	X	*TGA-MDRegLC*
Worm (THET), 2015	?	X	X	*ELC*
Baeyens, 2016	X	X	X	*PILC*
Pennell et al, 2016	√	X	X	*IDEAL-D*
Greenhalgh et al, 2017	√	√	√	*NASSS*
Gutiérrez-Ibarluzea, Chiumente & Dauben, 2017	X	X	X	*HTLC*
Hannan et al, 2017	√	?	?	*OIM-DA*
NASA, 2017	X	X	X	*PrLC*
Paris et al, 2017	X	?	√	*nHTLC4I*
Pecoraro & Luzi, 2017	X	X	√	*IRM-SaMDDP*
Meyer, Brühl & Omstad, 2018	X	X	X	*EUnetHTA-MDLC*
FDA, 2018	X	X	X	*FDA-MDRegLC*
Swissmedic, 2019	X	X	?	*Swissmedic-MDRegLC*
