# Supplementary material for: Lifecycle evaluation of medical devices: supporting or jeopardizing patient outcomes? A comparative analysis of evaluation models
Source: Int J Technol Assess Health Care. 2024 Jan 5;40(1):e2. doi: 10.1017/S026646232300274X (PMC10859834; doi:10.1017/S026646232300274X)
Supplement: Harkin et al. supplementary material 8 — Harkin et al. supplementary material [file S026646232300274Xsup008.pdf]

LIFECYCLE EVALUATION OF MEDICAL DEVICES – SUPPORTING OR JEOPARDIZING PATIENT OUTCOMES? A COMPARATIVE ANALYSIS OF EVALUATION MODELS

Authors: Kathleen Harkin, ORCID ID https://orcid.org/0000-0003-3260-9059; Jan Sorensen, ORCID ID https://orcid.org/0000-0003-0857-9267; Steve Thomas, ORCID ID https://orcid.org/0000-0001-9306-0114

Harkin_Supplemental-8_Model Characteristics

Model Characteristics

Model	**Device Type**	**Process Type**	**Evaluation Type**	**Model Type**	**Data Type**	**QA Score**	**Year 1st reference of model**
Baldock-NPD	Innovation – Product	A category of concepts that refer to actions of individuals or organisations	Prescriptive	Model	Qualitative	0	1960
DOI	Innovation	A logic used to explain a causal relationship in a variance theory	Exploratory and/or Explanatory	Theory	Quantitative	2	1962
PLC	Product	A sequence of events that describe how things change over time	Prescriptive	Model	Quantitative	1	1930
Bass	Product	A logic used to explain a causal relationship in a variance theory	Predictive	Model	Quantitative	2	1963
IRP	Innovation – Policy	A sequence of events that describe how things change over time	Exploratory and/or Explanatory	Model	Qualitative	1	1979
7Sm-IC	Innovation – Health Technology	A sequence of events that describe how things change over time	Descriptive	Model	Mixed	1	1981

BAH-NPD	Product	A category of concepts that refer to actions of individuals or organisations	Prescriptive	Model	Qualitative	0	1982
BLC	Business	Combines both a sequence of events that describe how things change over time and a category of concepts that refer to actions of individuals or organisations	Prescriptive	Framework	Qualitative	1	1982
ILC	Industry	A sequence of events that describe how things change over time	Exploratory and/or Explanatory	Model	Quantitative	2	1982
CK-NPD	Innovation – Product	A category of concepts that refer to actions of individuals or organisations	Prescriptive	Model	Qualitative	2	1980
Norton-Bass	Product	A logic used to explain a causal relationship in a variance theory	Predictive	Model	Quantitative	2	1987
SG-CK-NPD	Product	A category of concepts that refer to actions of individuals or organisations	Prescriptive	Model	Qualitative	2	1990
G-Bass-M	Product	A logic used to explain a causal relationship in a variance theory	Predictive	Model	Quantitative	2	1994
TRL	Technology	A sequence of events that describe how things change over time	Descriptive	Model	Qualitative	0	1995
MDDP	Medical Devices	A category of concepts that refer to actions of individuals or organisations	Prescriptive	Model	Qualitative	0	1997
4S-IEE	Health Technology	Combines both a sequence of events that describe how things change over time and a category of concepts that refer to actions of individuals or organisations	Prescriptive	Framework	Qualitative	0	1997

VA-NPD	Medical Devices – assistive	A category of concepts that refer to actions of individuals or organisations	Descriptive	Model	Qualitative	1	1997
TPLC	Medical Devices	A category of concepts that refer to actions of individuals or organisations	Descriptive	Framework	Qualitative	0	1999
RE-AIM	Health Technology – Public Health intervention	A category of concepts that refer to actions of individuals or organisations	Descriptive	Framework	Mixed	1	1999
TALC	Technology	A sequence of events that describe how things change over time	Prescriptive	Model	Quantitative	1	1991
MDLS	Medical Devices	A category of concepts that refer to actions of individuals or organisations	Prescriptive	Framework	Qualitative	1	1999
HCTLC	Medical Devices	A category of concepts that refer to actions of individuals or organisations	Descriptive	Framework	Qualitative	0	2003
SUHCD	Health Technology	A category of concepts that refer to actions of individuals or organisations	Prescriptive	Model	Qualitative	2	2004
DDDII	Innovation – Health Technology	A logic used to explain a causal relationship in a variance theory	Exploratory and/or Explanatory	Theory	Quantitative	3	2004
TALC-CAHF	Technology	A sequence of events that describe how things change over time	Predictive	Framework	Quantitative	2	2004
SG-MDDP	Medical Devices	A category of concepts that refer to actions of individuals or organisations	Descriptive	Model	Qualitative	1	2009

IEF	Industry	A sequence of events that describe how things change over time	Exploratory and/or Explanatory	Framework	Mixed	2	2009
IRM-TRL	Technology	A sequence of events that describe how things change over time	Descriptive	Model	Qualitative	0	2009
IDEAL	Innovation – Surgery	A category of concepts that refer to actions of individuals or organisations	Prescriptive	Framework	Qualitative	0	2009
EIM-2DA	Innovation – Surgery	A category of concepts that refer to actions of individuals or organisations	Prescriptive	Model	Qualitative	2	2010
TLC	Health Technology	A sequence of events that describe how things change over time	Descriptive	Model	Mixed	0	2010
IC+	Product	A logic used to explain a causal relationship in a variance theory	Descriptive	Model	Mixed	0	2010
MDLC	Medical Devices	A category of concepts that refer to actions of individuals or organisations	Prescriptive	Framework	Qualitative	0	2011
Bhuiyan-NPD	Innovation – Product	A category of concepts that refer to actions of individuals or organisations	Prescriptive	Framework	Qualitative	1	2011
USVP	Business	A sequence of events that describe how things change over time	Exploratory and/or Explanatory	Framework	Qualitative	2	2007
WW-IC	Innovation – Surgery	Combines both a sequence of events that describe how things change over time and a category of concepts that refer to actions of individuals or organisations	Prescriptive	Framework	Qualitative	0	2013

IC	Innovation – Product	Combines both a sequence of events that describe how things change over time and a category of concepts that refer to actions of individuals or organisations	Prescriptive	Model	Qualitative	0	2013
HCanada-MDRegLC	Health Technology	A category of concepts that refer to actions of individuals or organisations	Descriptive	Framework	Qualitative	0	2012
TGA-MDRegLC	Medical Devices	A category of concepts that refer to actions of individuals or organisations	Prescriptive	Framework	Qualitative	0	2014
RxLCF	Innovation – Surgery	A sequence of events that describe how things change over time	Prescriptive	Framework	Mixed	0	2014
PrLC	Technology	A sequence of events that describe how things change over time	Descriptive	Model	Qualitative	0	2014
ELC	Medical Devices – Equipment	A category of concepts that refer to actions of individuals or organisations	Prescriptive	Model	Qualitative	0	2005
IDEAL-D	Medical Devices	A category of concepts that refer to actions of individuals or organisations	Prescriptive	Framework	Qualitative	1	2016
PILC	Innovation – Product	A category of concepts that refer to actions of individuals or organisations	Descriptive	Framework	Qualitative	0	2016
nHTLC4I	Innovation – Health Technology	A category of concepts that refer to actions of individuals or organisations	Prescriptive	Model	Qualitative	1	2017
OIM-DA	Medical Devices – Orthopaedic implantables	A category of concepts that refer to actions of individuals or organisations	Prescriptive	Model	Qualitative	1	2017

HTLC	Health Technology	Combines both a sequence of events that describe how things change over time and a category of concepts that refer to actions of individuals or organisations	Prescriptive	Model	Qualitative	0	2017
NASSS	Innovation – Health Technology	A logic used to explain a causal relationship in a variance theory	Predictive	Theory	Quantitative	3	2017
IRM-SaMDDP	Medical Devices – Software	A category of concepts that refer to actions of individuals or organisations	Prescriptive	Framework	Qualitative	1	2014
EUnetHTA-MDLC	Health Technology	A category of concepts that refer to actions of individuals or organisations	Descriptive	Framework	Qualitative	0	2018
FDA-MDRegLC	Medical Devices	A category of concepts that refer to actions of individuals or organisations	Descriptive	Framework	Qualitative	0	2018
Swissmedic-MDRegLC	Medical Devices	A category of concepts that refer to actions of individuals or organisations	Prescriptive	Framework	Qualitative	0	2017
