# Supplementary material for: Lifecycle evaluation of medical devices: supporting or jeopardizing patient outcomes? A comparative analysis of evaluation models
Source: Int J Technol Assess Health Care. 2024 Jan 5;40(1):e2. doi: 10.1017/S026646232300274X (PMC10859834; doi:10.1017/S026646232300274X)
Supplement: Harkin et al. supplementary material 9 — Harkin et al. supplementary material [file S026646232300274Xsup009.pdf]

LIFECYCLE EVALUATION OF MEDICAL DEVICES – SUPPORTING OR JEOPARDIZING PATIENT OUTCOMES? A COMPARATIVE ANALYSIS OF EVALUATION MODELS

Authors: Kathleen Harkin, ORCID ID https://orcid.org/0000-0003-3260-9059; Jan Sorensen, ORCID ID https://orcid.org/0000-0003-0857-9267; Steve Thomas, ORCID ID https://orcid.org/0000-0001-9306-0114

Harkin_Supplemental-9_Study (reference texts) Characteristics

Table 9.1: Characteristics of the reference texts chosen to represent each model.docx

Model	Paper	Author/Proposer	Publication period	Geographical region	Publication Category	Funding/support or COI declared
Baldock-NPD	How to introduce a new product	Baldock	1960-1969	Americas	Conference/ Presentation	None declared
DOI	Diffusion of Innovations	Rogers	1960-1969	Americas	Book	None declared
PLC	Exploiting the Product Life Cycle	Levitt	1960-1969	Americas	Magazine	None declared
Bass	A new product growth for model consumer durables	Bass	1960-1969	Americas	Journal article	None declared
IRP	Life Histories of Innovations: How New Practices Become Routinized	Yin	1980-1989	Americas	Journal article	Funding/support declared
7Sm-IC	From "Promising Report" to "Standard Procedure": Seven Stages in the Career of a Medical Innovation	McKinlay	1980-1989	Americas	Journal article	Funding/support declared
BAH-NPD	New product management for the 1980's	Booz, Allen & Hamilton	1980-1989	Americas	Book	Uncertain
BLC	Stages of Growth	Galbraith	1980-1989	Americas	Journal article	Personal interests stated
ILC	Time Paths in the Diffusion of Product Innovations	Gort & Klepper	1980-1989	Americas	Journal article	Funding/support declared

CK-NPD	An Investigation into the New Product Process: Steps, Deficiencies, and Impact	Cooper & Kleinschmidt	1980-1989	Americas	Journal article	Funding/support declared
Norton-Bass	A diffusion theory model of adoption and substitution for successive generations of high-technology products	Norton & Bass	1980-1989	Americas	Journal article	None declared
SG-CK-NPD	Stage-gate systems: A new tool for managing new products	Cooper	1990-1999	Americas	Journal article	None declared
G-Bass-M	Why the Bass Model Fits without Decision Variables	Bass, Krishnan, & Jain	1990-1999	Americas	Journal article	None declared
TRL	Technology Readiness Levels. A White Paper	Mankins	1990-1999	Americas	Academic publication	None declared
MDDP	Design Control Guidance for Medical Device Manufacturers	FDA	1990-1999	Americas	Regulatory guidance	Funding/support declared
4S-IEE	The iterative use of economic evaluation as part of the process of health technology assessment	Sculpher, Buxton, & Drummond	1990-1999	Europe	Journal article	Funding/support declared
VA-NPD	The Department of Veterans Affairs Rehabilitation Research and Development Service's Technology Transfer Process	Sheredos & Cupo	1990-1999	Americas	Journal article	None declared
TPLC	Appendix D. Impact of the Regulatory Framework on Medical Device Development and Innovation. IN: Public Health Effectiveness of the FDA 510(k) Clearance Process	Feigal Jr. for the Institute of Medicine	2010-2019	Americas	Book	Funding/support declared
RE-AIM	Evaluating the Public Health Impact of Health Promotion Interventions: The RE-AIM Framework	Glasgow, Vogt, & Boles	1990-1999	Americas	Journal article	Funding/support declared
TALC	Crossing the Chasm	Moore	2000-2009	Americas	Book	Personal interests stated

MDLS	Medical Device Regulations. Global overview and guiding principles	Cheng	2000-2009	International	WHO Guidance	Funding/support declared
HCTLC	Medical Device Regulations. Global overview and guiding principles.	Cheng	2000-2009	International	WHO Guidance	Funding/support declared
SUHCD	Designing for Patient Safety: A Review of the Effectiveness of Design in the UK Health Service	Clarkson	2000-2009	Europe	Journal article	Funding/support declared
DDDII	Diffusion of Innovations in Service Organizations: Systematic Review and Recommendations	Greenhalgh et al	2000-2009	Americas	Journal article	Funding/support declared
TALC-CAHF	The technology adoption life cycle attractor: Understanding the dynamics of high-tech markets	Meade & Rabelo	2000-2009	Europe	Journal article	Funding/support declared
SG-MDDP	Stage-gate process for the development of medical devices	Pietzsch et al	2000-2009	International	Journal article	Funding/support declared
IEF	Developing a Framework for Mapping Industrial Emergence	Phaal et al	2000-2009	Americas	Conference/ Presentation	Funding/support declared
IRM-TRL	Technology readiness and risk assessments: A new approach	Mankins	2000-2009	Americas	Journal article	None declared
IDEAL	No surgical innovation without evaluation: the IDEAL recommendations	McCulloch et al	2000-2009	Europe	Journal article	Funding/support declared
EIM-2DA	EAES recommendations on methodology of innovation management in endoscopic surgery	Neugebauer & Becker et al	2010-2019	International	Journal article	Declared no COI
TLC	Introducing new technology safely	Mytton et al	2010-2019	Europe	Journal article	Funding/support declared
IC+	Innovate the Future: A Radical New Approach to IT Innovation	Croslin	2010-2019	Americas	Book	None declared

MDLC	Development of medical device policies	Velazquez-Berumen	2010-2019	International	WHO Guidance	Funding/support declared
Bhuiyan-NPD	A framework for successful new product development	Bhuiyan	2010-2019	Americas	Journal article	None declared
USVP	Understanding academic entrepreneurship: Exploring the emergence of university spin-off ventures using process theories	Rasmussen	2010-2019	Europe	Journal article	Funding/support declared
WW-IC	The Innovation Cycle: A Framework for Taking Surgical Innovation into Clinical Practice	Wright & Weinstein	2010-2019	Americas	Journal article	Declared no COI
IC	The Innovation Cycle	CIRAS	2010-2019	Americas	Website	None declared
HCanada-MDRegLC	Health Product Vigilance Framework. Release Date: 2012-09-12	Health Canada	2010-2019	Americas	Regulatory guidance	None declared
TGA-MDRegLC	Presentation: Life cycle of medical devices	Reeves & Garcia	2010-2019	Australia	Conference/ Presentation	Disclaimer
RxLCF	Beyond the dichotomy: a tool for distinguishing between experimental, innovative and established treatment	Provoost et al	2010-2019	Europe	Journal article	Funding/support declared
PrLC	NASA Systems Engineering Handbook Rev 2	NASA	2010-2019	Americas	Book	None declared
ELC	Managing the life cycle of medical equipment	Worm (THET)	2010-2019	International	Online resource	Funding/support declared
IDEAL-D	Adapting the IDEAL Framework and Recommendations for medical device evaluation: A modified Delphi survey	Pennell et al	2010-2019	International	Journal article	Funding/support declared
PILC	How the new Procurement Directive may contribute to spur innovative purchases in the health sector	Baeyens	2010-2019	Europe	Conference/ Presentation	None declared

nHTLC4I	New Health Technologies: Managing Access, Value and Sustainability	Paris et al	2010-2019	International	Book	Writing support acknowledged
OIM-DA	How should new orthopaedic implants be introduced: an example and recommendations for best practice	Hannan et al	2010-2019	Australia	Journal article	None declared
HTLC	The Life Cycle of Health Technologies. Challenges and Ways Forward	Gutiérrez-Ibarluzea, Chiumente & Dauben	2010-2019	International	Journal article	Declared no COI
NASSS	Beyond Adoption: A New Framework for Theorizing and Evaluating Nonadoption, Abandonment, and Challenges to the Scale-Up, Spread, and Sustainability of Health and Care Technologies	Greenhalgh et al	2010-2019	Europe	Journal article	Funding/support declared
IRM-SaMDDP	The integration of the risk management process with the lifecycle of medical device software	Pecoraro & Luzi	2010-2019	Europe	Journal article	None declared
EUnetHTA-MDLC	Session 3: Taking a lifecycle approach to EUnetHTA´s work: Current and future collaborations between early dialogue and Relative Effectiveness Assessments	Meyer, Brühl & Omstad	2010-2019	Europe	Conference/ Presentation	Funding/support declared
FDA-MDRegLC	TPLC - Total Product Life Cycle	FDA	2010-2019	Americas	Online resource	None declared
Swissmedic-MDRegLC	The tasks of Swissmedic - Lifecycle of a medical device	Swissmedic	2010-2019	Europe	Website	None declared
