# Supplementary material for: Lifecycle evaluation of medical devices: supporting or jeopardizing patient outcomes? A comparative analysis of evaluation models
Source: Int J Technol Assess Health Care. 2024 Jan 5;40(1):e2. doi: 10.1017/S026646232300274X (PMC10859834; doi:10.1017/S026646232300274X)
Supplement: Harkin et al. supplementary material 10 — Harkin et al. supplementary material [file S026646232300274Xsup010.pdf]

LIFECYCLE EVALUATION OF MEDICAL DEVICES – SUPPORTING OR JEOPARDIZING PATIENT OUTCOMES? A COMPARATIVE ANALYSIS OF EVALUATION MODELS

Authors: Kathleen Harkin, ORCID ID https://orcid.org/0000-0003-3260-9059; Jan Sorensen, ORCID ID https://orcid.org/0000-0003-0857-9267; Steve Thomas, ORCID ID https://orcid.org/0000-0001-9306-0114

Harkin_Supplemental-10_Lifecycle Evaluation Model Findings

Lifecycle Evaluation Model Findings

Model	**Publication Year***	**Purpose**	**Audience**	**Characterisation**	**Main interest**	**Outputs (Detailing ...)**	**Evaluation Timing**	**Scope - Lifecycle**	**Scope - Device**	**Model Archetype**
Baldock-NPD	1960	Define actions or activities	Trade & Industry	Development (stages)	What to do to develop a new product	'How to'	Repeated evaluation at each lifecycle stage	Provision	New	NPD
DOI	1962	Explore Influencing or impacted factors	Policymakers	Adoption (pattern)	Influencing factors	Influencing factors	Lifecycle evaluation at a single point in time	Acquisition & Utilisation	New	Diffusion
PLC	1965	Define actions or activities	Trade & Industry	Sales (pattern)	How to influence lifecycle	Influencing factors	Lifecycle evaluation at a single point in time	Provision	Established	Diffusion

Bass	1969	Describe the lifecycle	Trade & Industry	Sales (pattern)	Predicting sales/adoption	Influencing factors	Lifecycle evaluation at a single point in time	Provision	New	Diffusion
IRP	1981	Describe the lifecycle	Policymakers	Adoption (pattern)	Influencing factors	Influencing factors	Separate evaluation of different phases at a single timepoint	Utilisation	New	Diffusion
7Sm-IC	1981	Describe the lifecycle	Policymakers	Adoption (pattern)	Evidence levels	What happens	Separate evaluation of different phases at a single timepoint	Provision & Acquisition & Utilisation	New	Diffusion
BAH-NPD	1982	Define actions or activities	Trade & Industry	Development (stages)	What to do to develop a new product	'How to'	Repeated evaluation at each lifecycle stage	Provision	New	NPD
BLC	1982	Define actions or activities	Trade & Industry	Development (stages)	What to do to develop a new product	'How to'	Separate evaluation of different	Provision	New	NPD

							phases at a single timepoint			
ILC	1982	Describe the lifecycle	Trade & Industry	Development (pattern)	Influencing factors	Influencing factors	Lifecycle evaluation at a single point in time	Provision	New	Diffusion
CK-NPD	1986	Define actions or activities	Trade & Industry	Development (stages)	Performance evaluation	'How to'	Separate evaluation of different phases at a single timepoint	Provision	New	NPD
Norton-Bass	1987	Explore Influencing or impacted factors	Trade & Industry	Sales (pattern)	Influencing factors	Influencing factors	Lifecycle evaluation at a single point in time	Provision	New	Diffusion
SG-CK-NPD	1990	Determine readiness to progress	Trade & Industry	Development (stages)	Performance evaluation	'How to'	Repeated evaluation at each lifecycle stage	Provision	New	NPD

G-Bass-M	1994	Explore Influencing or impacted factors	Trade & Industry	Sales (pattern)	Influencing factors	Influencing factors	Lifecycle evaluation at a single point in time	Provision	All	Diffusion
TRL	1995	Determine readiness to progress	Trade & Industry	Development (stages)	Performance evaluation	Evaluation methods or activities	Repeated evaluation at each lifecycle stage	Provision & Acquisition	New	Hybrid
MDDP	1997	Define actions or activities	Trade & Industry	Development (stages)	Process controls	Regulation	Repeated evaluation at each lifecycle stage	Provision	All	NPD
4S-IEE	1997	Define actions or activities	Policymakers	Adoption (pattern)	Methods for outcomes evaluation	Evaluation methods or activities	Repeated evaluation at each lifecycle stage	Acquisition	New	Outcomes Evaluation
VA-NPD	1997	Determine readiness to progress	Policymakers	Development (stages)	What to do to develop a new product & Methods for	What happens	Repeated evaluation at each lifecycle stage	Provision & Acquisition & Utilisation	New	Hybrid*

					outcomes evaluation					
TPLC	2010	Define actions or activities	HC & General public	Development (stages)	Regulatory requirements & activities	Regulation	Repeated evaluation at each lifecycle stage	Provision & Utilisation	All	Hybrid
RE-AIM	1999	Define actions or activities	Policymakers	Adoption (pattern)	Methods for outcomes evaluation	Evaluation methods or activities	Repeated evaluation at each lifecycle stage	Provision & Acquisition & Utilisation	New	Outcomes Evaluation
TALC	2001	Define actions or activities	Trade & Industry	Sales (pattern)	How to influence lifecycle	Influencing factors	Lifecycle evaluation at a single point in time	Provision	New	Diffusion
MDLS	2003	Define actions or activities	Policymakers	Development (stages)	Regulatory requirements & activities	Regulation	Repeated evaluation at each lifecycle stage	Provision & Utilisation	All	Hybrid
HCTLC	2003	Define actions or activities	Policymakers	Development (stages)	What happens across the lifecycle	What happens	Lifecycle evaluation at a single	Provision & Acquisition	All	Hybrid

							point in time	& Utilisation		
SUHCD	2004	Define actions or activities	Healthcare	Development (stages)	What to do to develop a new product & Methods for outcomes evaluation	'How to'	Repeated evaluation at each lifecycle stage	Provision & Acquisition & Utilisation	New	Hybrid*
DDDII	2004	Explore Influencing or impacted factors	Policymakers	Adoption (pattern)	Influencing factors	Influencing factors	Lifecycle evaluation at a single point in time	Acquisition	New	Diffusion
TALC-CAHF	2004	Identify lifecycle stage	Trade & Industry	Sales (pattern)	Identifying where in the lifecycle a technology is	Evaluation methods or activities	Separate evaluation of different phases at a single timepoint	Provision	New	Diffusion
SG-MDDP	2009	Determine readiness to progress	Trade & Industry	Development (stages)	What to do to develop a new product	'How to'	Repeated evaluation at each lifecycle stage	Provision	New	NPD

IEF	2009	Describe the lifecycle	Trade & Industry	Development (pattern)	What happens across the lifecycle	Influencing factors	Separate evaluation of different phases at a single timepoint	Provision	New	Diffusion
IRM-TRL	2009	Determine readiness to progress	Trade & Industry	Development (stages)	Performance evaluation	Evaluation methods or activities	Repeated evaluation at each lifecycle stage	Provision & Acquisition	New	Hybrid
IDEAL	2009	Define actions or activities	Healthcare	Development (stages)	Methods for outcomes evaluation	Evaluation methods or activities	Repeated evaluation at each lifecycle stage	Provision & Acquisition & Utilisation	New	Outcomes Evaluation
EIM-2DA	2010	Identify lifecycle stage	Healthcare	Development (stages)	Methods for outcomes evaluation	Evaluation methods or activities	Repeated evaluation at each lifecycle stage	Provision & Acquisition & Utilisation	New	Outcomes Evaluation
TLC	2010	Describe the lifecycle	Healthcare	Adoption (pattern)	What happens across the lifecycle	What happens	Separate evaluation of different phases at a	Provision & Acquisition	All	Outcomes Evaluation

							single timepoint	& Utilisation		
IC+	2010	Explore Influencing or impacted factors	Trade & Industry	Sales (pattern)	What happens across the lifecycle	Evaluation methods or activities	Separate evaluation of different phases at a single timepoint	Provision	New	Diffusion
MDLC	2011	Define actions or activities	Policymakers	Development (stages)	Process controls	'How to'	Separate evaluation of different phases at a single timepoint	Provision & Acquisition & Utilisation & Regulation	All	Hybrid*
Bhuiyan-NPD	2011	Define actions or activities	Trade & Industry	Development (stages)	Performance evaluation	'How to'	Repeated evaluation at each lifecycle stage	Provision	New	NPD
USVP	2011	Describe the lifecycle	Trade & Industry	Development (stages)	Influencing factors	Influencing factors	Lifecycle evaluation at a single point in time	Provision	New	NPD

WW-IC	2013	Define actions or activities	Healthcare	Development (stages)	Methods for outcomes evaluation	Evaluation methods or activities	Repeated evaluation at each lifecycle stage	Provision & Acquisition & Utilisation	New	Outcomes Evaluation
IC	2013	Define actions or activities	Trade & Industry	Development (stages)	Performance evaluation	'How to'	Repeated evaluation at each lifecycle stage	Provision	New	NPD
HCanada-MDRegLC	2013	Define actions or activities	General public	Development (stages)	Regulatory requirements & activities	Regulation	Repeated evaluation at each lifecycle stage	Provision & Utilisation	All	Hybrid
TGA-MDRegLC	2014	Define actions or activities	Trade & Industry	Development (stages)	Regulatory requirements & activities	Regulation	Repeated evaluation at each lifecycle stage	Provision & Utilisation	All	Hybrid
RxLCF	2014	Identify lifecycle stage	Healthcare	Development (stages)	Identifying where in the lifecycle a technology is	Evaluation methods or activities	Repeated evaluation at each lifecycle stage	Acquisition	New	Outcomes Evaluation

PrLC	2017	Define actions or activities	Policymakers	Development (stages)	What to do to develop a new product & Methods for outcomes evaluation	‘How to’	Repeated evaluation at each lifecycle stage	Provision & Acquisition & Utilisation	New	Hybrid*
ELC	2015	Define actions or activities	Healthcare	Health technology management activities	How to perform HTM (highlighting potentially false assumptions & mitigating actions)	Evaluation methods or activities	Repeated evaluation at each lifecycle stage	Acquisition & Utilisation	Established	Diffusion
IDEAL-D	2016	Define actions or activities	Regulators	Development (stages)	Methods for outcomes evaluation	Evaluation methods or activities	Repeated evaluation at each lifecycle stage	Provision & Acquisition & Utilisation	New	Outcomes Evaluation
PILC	2016	Define actions or activities	T&I and HC	Development (stages)	Approaches to integrating innovation into the healthcare system	Regulation	Repeated evaluation at each lifecycle stage	Provision & Acquisition	New	Diffusion

nHTLC4I	2017	Define actions or activities	Policymakers	Development (stages)	Approaches to integrating innovation into the healthcare system	Regulation	Repeated evaluation at each lifecycle stage	Acquisition & Utilisation	New	Diffusion
OIM-DA	2017	Define actions or activities	Healthcare	Development (stages)	Methods for outcomes evaluation	Evaluation methods or activities	Repeated evaluation at each lifecycle stage	Acquisition & Utilisation	New	Outcomes Evaluation
HTLC	2017	Define actions or activities	Policymakers	Development (stages)	Methods for outcomes evaluation	Evaluation methods or activities	Repeated evaluation at each lifecycle stage	Provision & Acquisition & Utilisation	New	Outcomes Evaluation
NASSS	2017	Explore Influencing or impacted factors	Policymakers	Adoption (pattern)	Predicting sales/adoption	Evaluation methods or activities	Separate evaluation of different phases at a single timepoint	Acquisition & Utilisation	New	Diffusion
IRM-SaMDDP	2017	Define actions or activities	Trade & Industry	Development (stages)	What to do to develop a new product	'How to'	Separate evaluation of different	Provision	New	NPD

							phases at a single timepoint			
EUnetHTA-MDLC	2018	Define actions or activities	T&I and HTA	Adoption (pattern)	Methods for outcomes evaluation	Evaluation methods or activities	Repeated evaluation at each lifecycle stage	Provision & Acquisition	New	Outcomes Evaluation
FDA-MDRegLC	2018	Define actions or activities	General public	Development (stages)	Regulatory requirements & activities	Regulation	Repeated evaluation at each lifecycle stage	Provision & Utilisation	All	Hybrid
Swissmedic-MDRegLC	2019	Define actions or activities	T&I and General public	Development (stages)	Regulatory requirements & activities	Regulation	Repeated evaluation at each lifecycle stage	Provision & Utilisation	All	Hybrid

Note: Hybrid* indicates that the model includes a focus on outcomes evaluation as well as either new product development (NPD) and/or diffusion.
